# Psilacetin derivatives: fumarate salts of the meth­yl–ethyl, meth­yl–allyl and diallyl variants of the psilocin prodrug

**DOI:** 10.1107/S2056989021000116

**Published:** 2021-01-08

**Authors:** Duyen N. K. Pham, Andrew R. Chadeayne, James A. Golen, David R. Manke

**Affiliations:** a University of Massachusetts Dartmouth, 285 Old Westport Road, North Dartmouth, MA 02747, USA; b CaaMTech, Inc., 58 East Sunset Way, Suite 209, Issaquah, WA 98027, USA

**Keywords:** crystal structure, tryptamines, indoles, fumarates, hydrogen bonding

## Abstract

The solid-state structures of the salts of three psilacetin derivatives, namely, 4-acet­oxy-*N*-eth­yl-*N*-methyl­tryptammonium (4-AcO-MET) hydro­fumarate {sys­tematic name: [2-(4-acet­yloxy-1*H*-indol-3-yl)eth­yl](meth­yl)ethyl­aza­nium 3-carb­oxy­prop-2-enoate}, C_15_H_21_N_2_O_2_^+^·C_4_H_3_O_4_^−^, 4-acet­oxy-*N*-allyl-*N*-methyl­tryptammonium (4-AcO-MALT) hydro­fumarate {systematic name: [2-(4-acet­yl­oxy-1*H*-indol-3-yl)eth­yl](meth­yl)prop-2-enyl­aza­nium 3-carb­oxy­prop-2-eno­ate}, C_16_H_21_N_2_O_2_^+^·C_4_H_3_O_4_^−^, and 4-acet­oxy-*N*,*N*-di­allyl­tryptammonium (4-AcO-DALT) fumarate–fumaric acid (1/1) (systematic name: bis­{[2-(4-acet­yloxy-1*H*-indol-3-yl)eth­yl]diprop-2-enyl­aza­nium} but-2-enedioate–(*E*)-butenedioic acid (1/1)), 2C_18_H_23_N_2_O_2_^+^·C_4_H_2_O_4_^2−^·C_4_H_4_O_4_, are reported. All three salts possess a protonated tryptammonium cation. The 4-AcO-MET and 4-AcO-MALT compounds are charge-balanced by 3-carb­oxy­acrylate (hydro­fumarate) anions. The 4-AcO-DALT complex crystallizes as a two-to-one tryptammonium-to-fumarate salt, which co-crystallizes with a fumaric acid mol­ecule. Each structure is consolidated by N—H⋯O and O—H⋯O hydrogen bonds.

## Abstract 2

The crystal structures of the fumarate salts of three prodrugs of synthetic psychedelics (4-AcO-MET, 4-AcO-MALT and 4-AcO-DALT) are reported.

## Chemical context   

Psychotropic tryptamines have emerged as leading candidates in the treatment of mood disorders, including anxiety, addiction, depression and post-traumatic stress disorder (Byock, 2018 ▸; Daniel & Haberman, 2017 ▸). Perhaps the best known of these tryptamines is psilocybin, *N*,*N*,*N*-trimethyl-4-phospho­r­yloxytryptamine (C_12_H_17_N_2_O_4_P), which has recently been cleared for a number of clinical trials after receiving the ‘breakthrough therapy’ designation from the US Food and Drug Administration (Feltman, 2019 ▸). When psilocybin is consumed orally, it is hydrolysed to generate 4-hy­droxy-*N*,*N*-di­methyl­tryptamine, C_12_H_16_N_2_O (4-HO-DMT), or psilocin, which is the active metabolite. Psilocin is a potent serotonin 2a agonist, and is the primary origin of its psychoactive properties (Geiger *et al.*, 2018 ▸).

Psilacetin, 4-acet­oxy-*N*,*N*-di­methyl­tryptamine, C_14_H_18_N_2_O_2_ (4-AcO-DMT), is a synthetic alternative to psilocybin. It also acts as a prodrug of psilocin, with the acetyl group of psilacetin being hydrolysed as it is metabolized, converting 4-AcO-DMT to 4-HO-DMT. Psilacetin is easier to synthesize than psilocybin, and can also be produced at a lower cost, making it, perhaps, a better candidate for the delivery of psilocin (Nichols & Frescas, 1999 ▸). Presumably, all 4-acet­oxy-substituted tryptamines would similarly function as prodrugs for their active metabolite psilocin analogues. Three such compounds are 4-acet­oxy-*N*-ethyl-*N*-methyl­tryptamine, C_15_H_20_N_2_O_2_ (4-AcO-MET), 4-acet­oxy-*N*-allyl-*N*-methyl­tryptamine, C_16_H_20_N_2_O_2_ (4-AcO-MALT), and 4-acet­oxy-*N*,*N*-di­allyl­tryptamine, C_18_H_22_N_2_O_2_ (4-AcO-DALT). These variations of psilacetin have garnered very little attention in the scientific literature, with only one reference made to 4-AcO-MET in a chromatographic screening article prior to this year (Lehmann *et al.*, 2017 ▸). A recent report on the activity of psilacetin analogues and their metabolites included 4-AcO-MET (Klein *et al.*, 2020 ▸). Herein, we report the first solid-state structures of the fumarate salts of 4-AcO-MET, 4-AcO-MALT and 4-AcO-DALT.
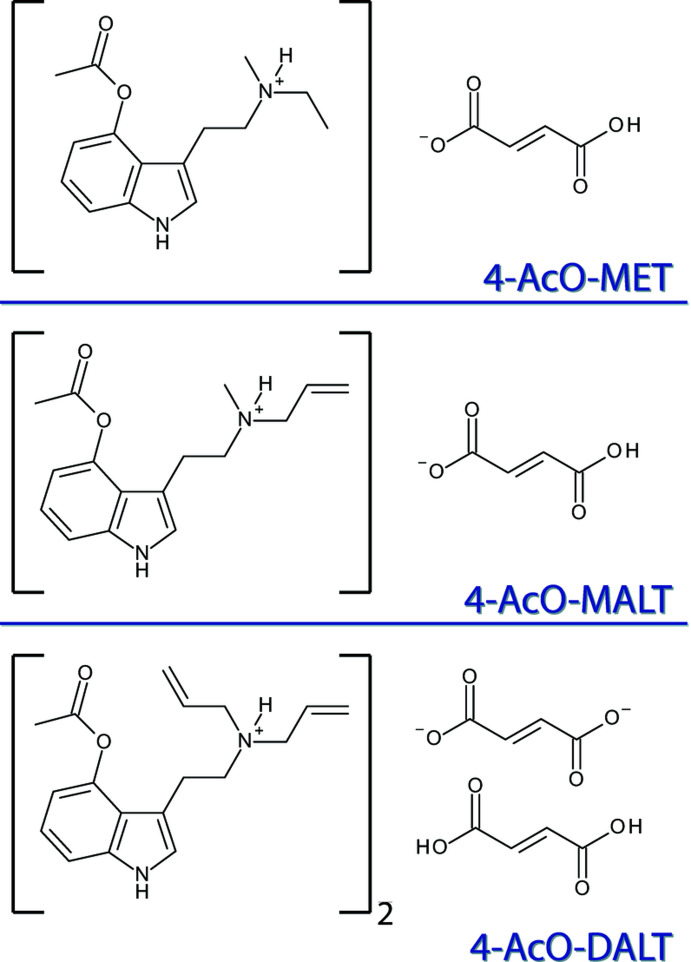


## Structural commentary   

**Figure 1:**
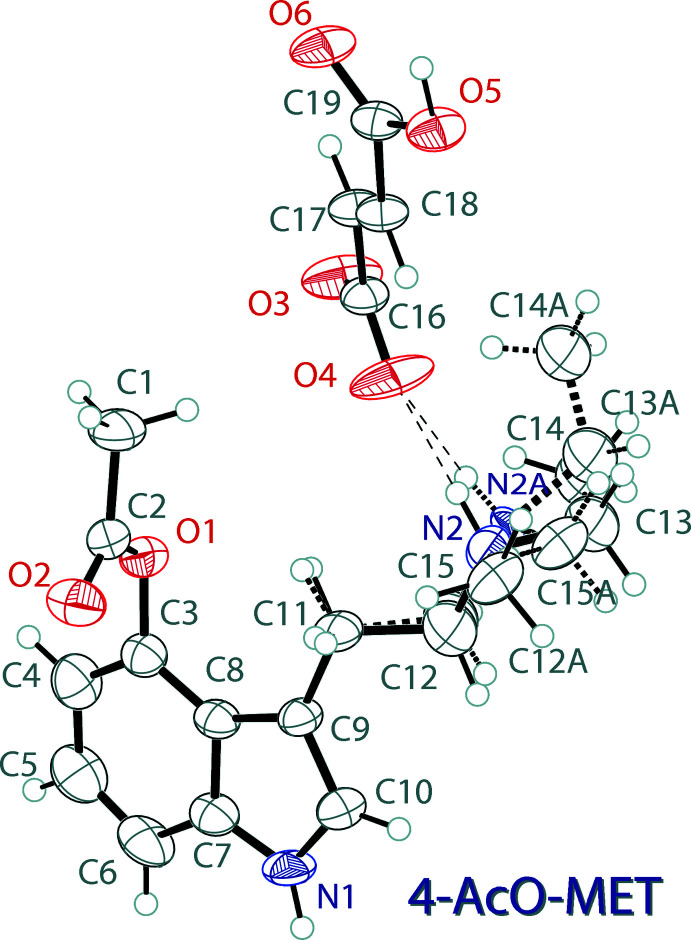
The mol­ecular structure of 4-AcO-MET hydro­fumarate (I), showing the atomic labeling. Displacement ellipsoids are drawn at the 50% probability level. Dashed bonds indicate a disordered component in the structures. Hydrogen bonds are shown as dashed lines.

The mol­ecular structure of 4-AcO-MET hydro­fumarate, (I), is shown in Fig. 1 ▸. The asymmetric unit contains one 4-acet­oxy-*N*-ethyl-*N*-methyl­tryptammonium (C_15_H_21_N_2_O_2_^+^) cation and one hydro­fumarate (C_4_H_3_O_4_^−^) anion. The indole ring system of the cation is near planar with an r.m.s. deviation of 0.015 Å. The hydro­fumarate anion is slightly twisted, demonstrating a deviation from planarity of 0.158 Å, and a C16/O3/O4 carboxyl­ate to C19/O5/O6 carb­oxy­lic acid plane normal angle of 23.0 (3)°. The *N*-methyl-*N*-ethyl group of the cation is disordered over two orientations in a 0.76 (1):0.24 (7) ratio. The ethyl­ammonium arm is turned slightly away from the plane of the indole, with C10—C9—C11—C12 and C10—C9—C11—C12*A* torsion angles of 39.7 (7) and 49.5 (2)°, respectively, for the two orientations.

**Figure 2:**
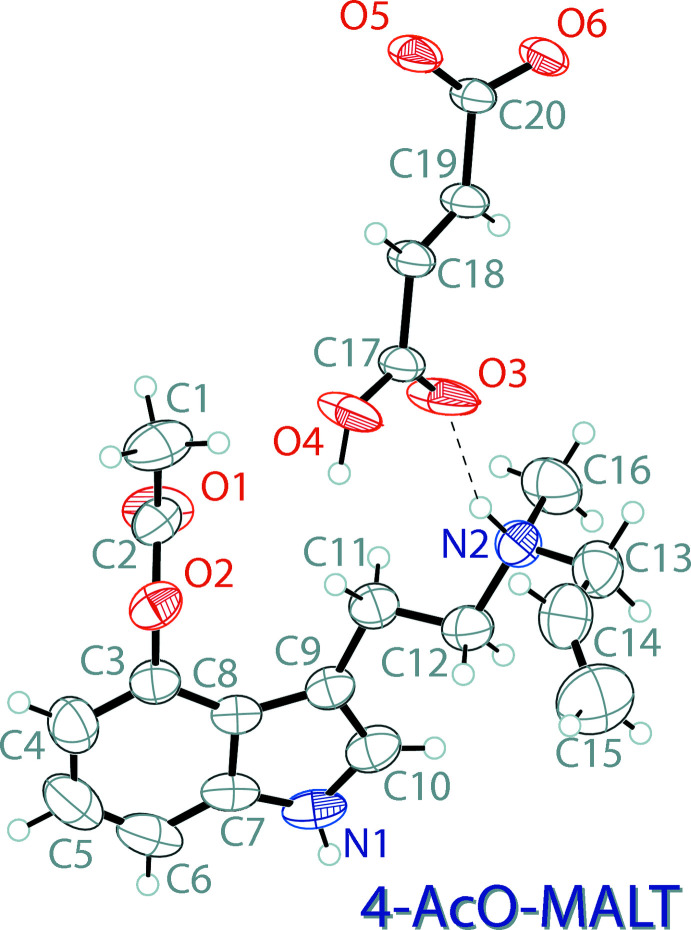
The mol­ecular structure of 4-AcO-MALT hydro­fumarate (II), showing the atomic labeling. Displacement ellipsoids are drawn at the 50% probability level. Hydrogen bonds are shown as dashed lines.

The mol­ecular structure of 4-AcO-MALT hydro­fumarate, (II), is shown in Fig. 2 ▸. The asymmetric unit contains one 4-acet­oxy-*N*-allyl-*N*-methyl­tryptammonium (C_16_H_21_N_2_O_2_^+^) cation and one hydro­fumarate (C_4_H_3_O_4_^−^) anion. The indole ring system of the compound is almost planar with an r.m.s. deviation from planarity of 0.006 Å. The ethyl­ammonium arm is turned slightly away from the plane of the indole ring, with a C10—C9—C11—C12 torsion angle of 39.8 (4)°. The hydro­fumarate anion is slightly twisted, showing a deviation from planarity of 0.128 Å, and a C20/O5/O6 carboxyl­ate to C17/O3/O4 carb­oxy­lic acid twist of 18.6 (2)°.

**Figure 3:**
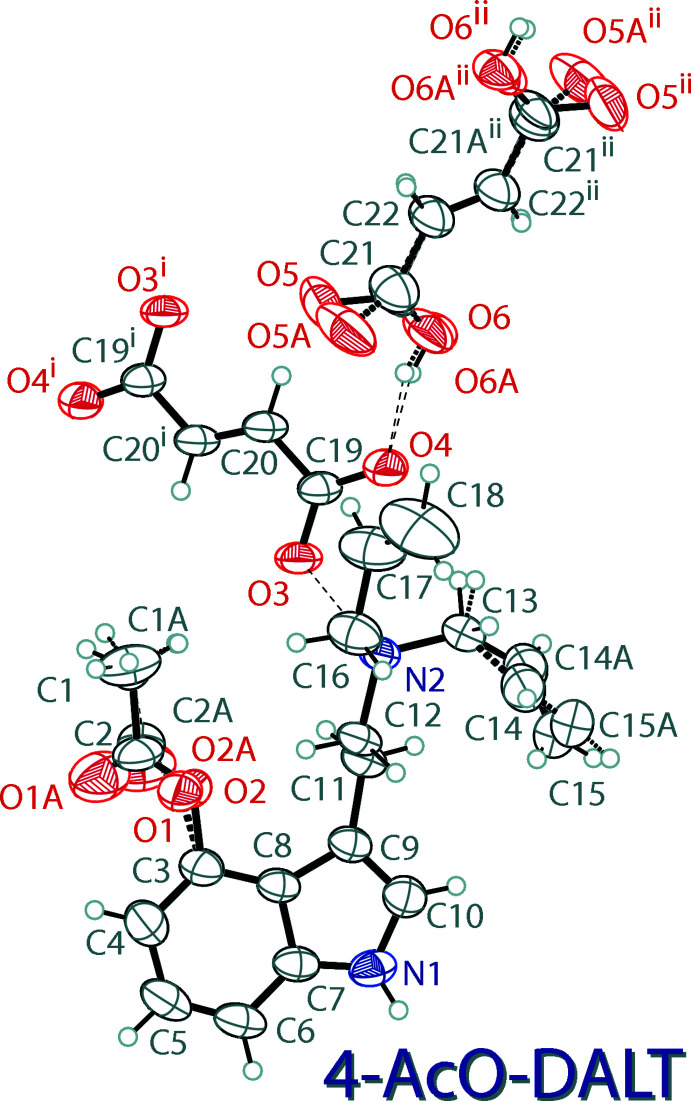
The mol­ecular structure of 4-AcO-DALT fumarate fumaric acid (III), showing the atomic labeling. Displacement ellipsoids are drawn at the 50% probability level. Dashed bonds indicate a disordered component in the structures. Hydrogen bonds are shown as dashed lines. Symmetry codes: (i) 1 − *x*, 2 − *y*, 1 − *z*; (ii) 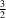 − *x*, 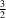 − *y*, 1 − *z*.

The mol­ecular structure of 4-AcO-DALT fumarate–fumaric acid, (III), is shown in Fig. 3 ▸. The asymmetric unit contains one 4-acet­oxy-*N*,*N*-di­allyl­tryptammonium (C_18_H_23_N_2_O_2_^+^) cation, one half of a fumarate (C_2_HO_2_^−^) dianion, and one half of a fumaric acid (C_2_H_4_O_2_) mol­ecule. The indole ring system of the compound is near planar with a r.m.s. deviation from planarity of 0.016 Å. The ethyl­ammonium arm is turned significantly away from the plane of the indole ring, with a C10—C9—C11—C12 torsion angle of 104.3 (2)°. The complete fumarate dianion is generated through crystallographic inversion symmetry, and is also near planar, with an r.m.s. deviation from planarity of 0.004 Å. The full disordered (*vide infra*) fumaric acid mol­ecule is generated through inversion, and also demonstrates near planarity, with r.m.s. deviations from planarity of 0.082 and 0.083 Å for the two conformations. One of the allyl groups in the cation is disordered over two orientations with a 0.90 (1):0.10 (1) ratio. The fumaric acid mol­ecule is also disordered over two components with a 0.52 (4):0.48 (4) ratio. The 4-acet­oxy group also shows a disorder over two orientations with a 0.62 (4):0.38 (4) ratio. The carboxyl­ate group of the fumarate anion is delocalized, with C—O distances of 1.251 (3) and 1.258 (2) Å.

## Supra­molecular features   

**Figure 4:**
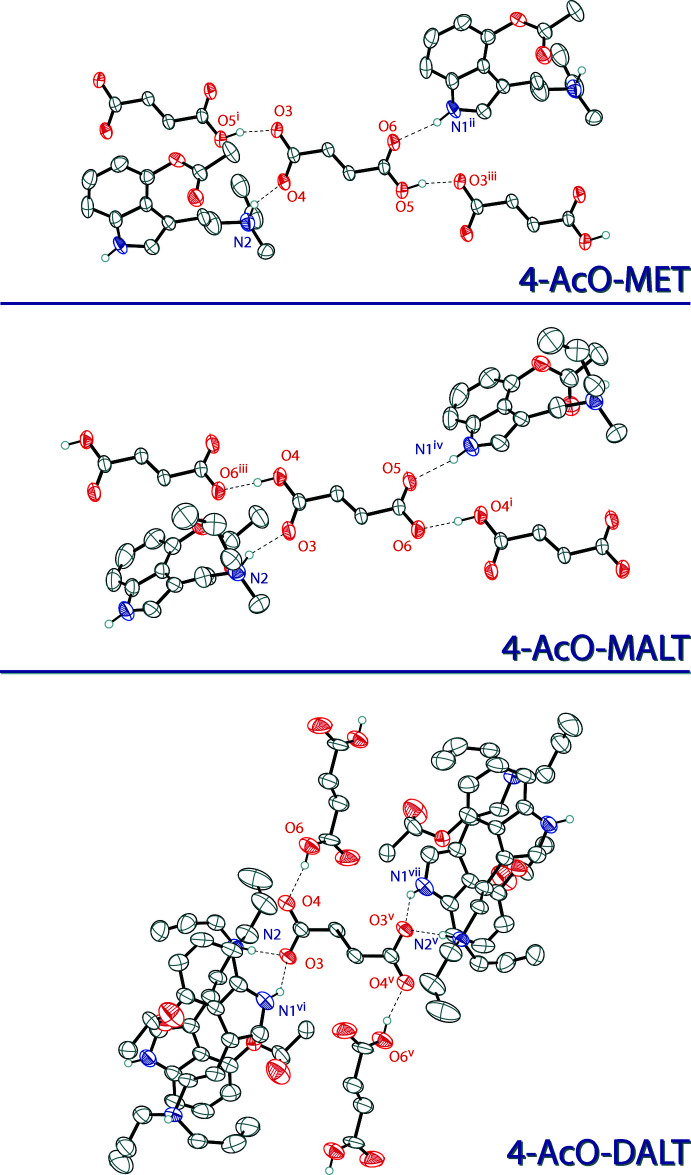
The hydrogen bonding environments of the hydro­fumarate ion in the structure of (I) (top), the hydro­fumarate ion in the structure of (II) (middle), and the fumarate dianion in the structure of (III) (bottom). Displacement ellipsoids are drawn at the 50% probability level. Hydrogen atoms not involved in hydrogen bonds are omitted for clarity. Only one component of disorders are shown. Symmetry codes: (i) 1 + *x*, *y*, *z*; (ii) −1 + *x*, *y*, 1 + *z*; (iii) −1 + *x*, *y*, *z*; (iv) 2 + *x*, *y*, 1 + *z*; (v) 1 − *x*, 2 − *y*, 1 − *z*; (vi) 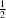 − *x*, 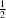 + *y*, 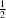 − *z*; (vii) 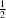 + *x*, 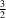 − *y*, 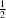 + *z*.

**Figure 5:**
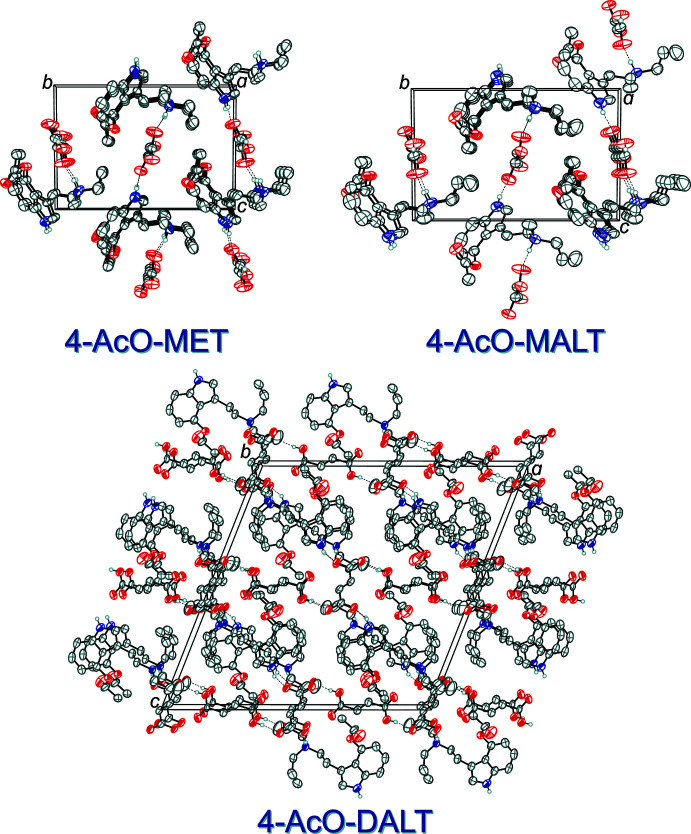
The crystal packing of (I) (top left), viewed along the *a*-axis direction, the crystal packing of (II) (top right), viewed along the *a*-axis direction and the crystal packing of (III) (bottom), viewed along the *b*-axis direction. The hydrogen bonds (Tables 1 ▸–3 ▸ ▸) are shown as dashed lines. Displacement ellipsoids are drawn at the 50% probability level. Hydrogen atoms not involved in hydrogen bonds are omitted for clarity. Only one component of disorders are shown.

In the extended structure of (I), the *N*-ethyl-*N*-methyl­tryptammonium cations and hydro­fumarate anions are linked together in a two-dimensional network lying in the (010) plane through N—H⋯O and O—H⋯O hydrogen bonds (Table 1 ▸). The O—H group of the hydro­fumarate hydrogen bonds with the carbonyl oxygen atom of the carboxyl­ate unit of another hydro­fumarate ion, the ammonium N—H hydrogen bonds to the negatively charged oxygen atom of the carboxyl­ate group of a hydro­fumarate ion, and the indole N—H hydrogen bonds to the carbonyl oxygen atom of the carb­oxy­lic acid unit of a hydro­fumarate ion (Fig. 4 ▸, top). The packing of 4-AcO-MET hydro­fumarate is shown at the top left of Fig. 5 ▸.

In the extended structure of (II), the *N*-allyl-*N*-methyl­tryptammonium cations and hydro­fumarate anions are linked together in an infinite two-dimensional network parallel to (010) through N—H⋯O and O—H⋯O hydrogen bonds (Table 2 ▸). The O—H group of the hydro­fumarate hydrogen bonds with the negatively charged oxygen atom of the carboxyl­ate unit of another hydro­fumarate ion, the indole N—H hydrogen bond to the carbonyl O atom of the carboxyl­ate group of the hydro­fumarate ion, and the ammonium N—H hydrogen bonds to the carbonyl oxygen atom of the carb­oxy­lic acid unit of the hydro­fumarate ion (Fig. 4 ▸, center). The packing of 4-AcO-MALT hydro­fumarate is shown at the top right of Fig. 5 ▸.

In the extended structure of (III), the *N,N*-di­allyl­tryptammonium cations, fumarate dianions and fumaric acid mol­ecules are linked together in a three-dimensional network through N—H⋯O and O—H⋯O hydrogen bonds (Table 3 ▸). The O—H group of the fumaric acid, the ammonium N—H, and the indole N—H group all hydrogen bond to oxygen atoms of the fumarate dianion (Fig. 4 ▸, bottom). The packing of 4-AcO-DALT fumarate–fumaric acid is shown at the bottom of Fig. 5 ▸.

## Database survey   

The three structures reported here are closely related to psilacetin, which has been reported as both the hydro­fumarate (HOCJUH: Chadeayne, Golen & Manke 2019*b* ▸) and fumarate (XOFDOO: Chadeayne *et al.*, 2019*a* ▸) salts. 4-AcO-MET and 4-AcO-MALT both form hydro­fumarate salts, though the hydrogen-bonding networks vary from that observed for psilacetin. 4-AcO-DALT crystallizes as the fumarate salt, but also co-crystallizes with a fumaric acid mol­ecule in the structure. The structure of the acet­oxy-protected version of the active metabolite of aeruginascin, 4-acet­oxy-*N*,*N*,*N*-tri­methyl­tryptamine, has been reported (XUXDUS: Chadeayne, Pham, Reid *et al.*, 2020 ▸). The other reported structures of tryptammonium hydro­fumarate monoanion salts are for 4-hy­droxy-*N*-methyl-*N*-iso­propyl­tryptamine and *N*-methyl-*N*-iso­propyl­tryptamine (RONSUL and RONSOF: Chadeayne, Pham *et al.*, 2019*b* ▸) and *N*-ethyl-*N*-*n*-propyl­tryptamine and *N*-allyl-*N*-methyl­tryptamine (CCDC 2012495 and CCDC 2012494: Chadeayne *et al.*, 2020*c* ▸). The other reported structures of tryptammonium fumarate dianion salts are for 4-hy­droxy-*N*,*N*-di­propyl­tryptamine (CCDC 1962339: Chadeayne *et al.*, 2019*b* ▸), 4-hy­droxy-*N*-methyl-*N*-iso­propyl­tryptamine (CCDC 1987588: Chadeayne *et al.*, 2020*a* ▸) and 4-hy­droxy-*N*-methyl­tryptamine (CCDC 1992278: Chadeayne *et al.*, 2020*b* ▸).

## Synthesis and crystallization   

Single crystals of 4-acet­oxy-*N*-ethyl-*N*-methyl­tryptammonium hydro­fumarate suitable for X-ray analysis were obtained from the slow evaporation of an ethano­lic solution of a commercial sample (The Indole Shop). A commercial sample of 4-acet­oxy-*N*-allyl-*N*-methyl­tryptammonium hydro­fumarate (The Indole Shop) was recrystallized by the slow evaporation of an aqueous solution to yield samples suitable for single crystal X-ray diffraction studies. Single crystals of bis­(4-acet­oxy-*N*,*N*-di­allyl­tryptammonium) fumarate fumaric acid suitable for X-ray analysis were obtained from the slow evaporation of an acetone solution of a commercial sample (The Indole Shop).

## Refinement   

Crystal data, data collection and structure refinement details are summarized in Table 4 ▸. O and N-bound H atoms were refined with the restraints O—H = 0.88±1 and N—H = 0.87±1 Å and with *U*_iso_(H) = 1.2*U*_eq_(N) or 1.5*U*_eq_(O). C-bound H atoms were positioned geometrically and refined using a riding model: C—H = 0.95 Å with *U*_iso_(H) = 1.2*U*_eq_(C) or 1.5*U*_eq_(C-meth­yl).

## Supplementary Material

Crystal structure: contains datablock(s) I, II, III, global. DOI: 10.1107/S2056989021000116/hb7959sup1.cif

Structure factors: contains datablock(s) I. DOI: 10.1107/S2056989021000116/hb7959Isup2.hkl

Supporting information file. DOI: 10.1107/S2056989021000116/hb7959Isup5.cml

Structure factors: contains datablock(s) II. DOI: 10.1107/S2056989021000116/hb7959IIsup3.hkl

Supporting information file. DOI: 10.1107/S2056989021000116/hb7959IIsup6.cml

Structure factors: contains datablock(s) III. DOI: 10.1107/S2056989021000116/hb7959IIIsup4.hkl

Supporting information file. DOI: 10.1107/S2056989021000116/hb7959IIIsup7.cml

CCDC references: 2053845, 2053844, 2053843

Additional supporting information:  crystallographic information; 3D view; checkCIF report

## Figures and Tables

**Table 1:** Hydrogen-bond geometry (Å, °) for (I)

*D*—H⋯*A*	*D*—H	H⋯*A*	*D*⋯*A*	*D*—H⋯*A*
N1—H1⋯O6^i^	0.86 (1)	2.02 (2)	2.858 (4)	165 (5)
N2—H2⋯O4	0.88 (2)	1.85 (4)	2.644 (6)	150 (7)
N2*A*—H2*A*⋯O4	0.87 (2)	1.94 (6)	2.776 (14)	162 (17)
O5—H5*A*⋯O3^ii^	0.89 (2)	1.61 (2)	2.459 (4)	160 (6)

Symmetry codes: (i) 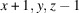; (ii) 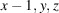.

**Table 2:** Hydrogen-bond geometry (Å, °) for (II)

*D*—H⋯*A*	*D*—H	H⋯*A*	*D*⋯*A*	*D*—H⋯*A*
N1—H1⋯O5^i^	0.87 (1)	2.00 (2)	2.857 (3)	169 (4)
O4—H4*A*⋯O6^ii^	0.89 (1)	1.56 (2)	2.454 (3)	175 (6)

Symmetry codes: (i) 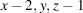; (ii) 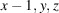.

**Table 3:** Hydrogen-bond geometry (Å, °) for (III)

*D*—H⋯*A*	*D*—H	H⋯*A*	*D*⋯*A*	*D*—H⋯*A*
N1—H1⋯O3^i^	0.87 (1)	2.22 (2)	2.962 (2)	144 (2)
N2—H2⋯O3	0.88 (1)	1.87 (1)	2.7446 (19)	177 (2)
O6—H6*A*⋯O4	0.82	1.72	2.530 (17)	168
O6*A*—H6*AA*⋯O4	0.82	1.81	2.577 (16)	156

Symmetry code: (i) 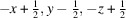.

**Table 4:** Experimental details

	(I)	(II)	(III)
Crystal data			
Chemical formula	C_15_H_21_N_2_O_2_^+^·C_4_H_3_O_4_^−^	C_16_H_21_N_2_O_2_^+^·C_4_H_3_O_4_^−^	2C_18_H_23_N_2_O_2_^+^·C_4_H_2_O_4_^2−^·C_4_H_4_O_4_
*M*_r_	376.40	388.41	828.90
Crystal system, space group	Monoclinic, *P*2_1_	Monoclinic, *P*2_1_	Monoclinic, *C*2/*c*
Temperature (K)	200	297	297
*a*, *b*, *c* (Å)	7.9555 (4), 13.3696 (7), 9.9708 (5)	7.9702 (4), 14.1788 (7), 9.8035 (5)	23.6642 (19), 8.4204 (7), 23.4002 (18)
β (°)	112.874 (2)	113.394 (2)	111.614 (2)
*V* (Å^3^)	977.12 (9)	1016.80 (9)	4334.9 (6)
*Z*	2	2	4
Radiation type	Mo *K*α	Mo *K*α	Mo *K*α
μ (mm^−1^)	0.10	0.09	0.09
Crystal size (mm)	0.24 × 0.2 × 0.2	0.34 × 0.24 × 0.2	0.22 × 0.2 × 0.12

Data collection			
Diffractometer	Bruker D8 Venture CMOS	Bruker D8 Venture CMOS	Bruker D8 Venture CMOS
Absorption correction	Multi-scan (*SADABS*; Bruker, 2018 ▸)	Multi-scan (*SADABS*; Bruker, 2018 ▸)	Multi-scan (*SADABS*; Bruker, 2018 ▸)
*T*_min_, *T*_max_	0.708, 0.745	0.686, 0.745	0.715, 0.745
No. of measured, independent and observed [*I* > 2σ(*I*)] reflections	21973, 3536, 3232	26393, 3797, 3516	99597, 4126, 3441
*R*_int_	0.036	0.039	0.040
(sin θ/λ)_max_ (Å^−1^)	0.603	0.610	0.611

Refinement			
*R*[*F* ^2^ > 2σ(*F* ^2^)], *wR*(*F* ^2^), *S*	0.052, 0.146, 1.04	0.043, 0.113, 1.04	0.051, 0.140, 1.06
No. of reflections	3536	3797	4126
No. of parameters	271	264	354
No. of restraints	15	4	114
H-atom treatment	H atoms treated by a mixture of independent and constrained refinement	H atoms treated by a mixture of independent and constrained refinement	H atoms treated by a mixture of independent and constrained refinement
Δρ_max_, Δρ_min_ (e Å^−3^)	0.39, −0.55	0.27, −0.16	0.50, −0.30
Absolute structure	Flack *x* determined using 1411 quotients [(*I* ^+^)−(*I* ^−^)]/[(*I* ^+^)+(*I* ^−^)] (Parsons *et al.*, 2013 ▸)	Flack *x* determined using 1577 quotients [(*I* ^+^)−(*I* ^−^)]/[(*I* ^+^)+(*I* ^−^)] (Parsons *et al.*, 2013 ▸)	–
Absolute structure parameter	−0.3 (3)	0.4 (3)	–

Computer programs: *APEX3* and *SAINT* (Bruker, 2018 ▸), *SHELXT2014* (Sheldrick, 2015*a* ▸), *SHELXL2018* (Sheldrick, 2015*b* ▸), *OLEX2* (Dolomanov *et al.*, 2009 ▸), and *publCIF* (Westrip, 2010 ▸).

## supplementary crystallographic information

### [2-(4-Acetyloxy-1H-indol-3-yl)ethyl](methyl)ethylazanium 3-carboxyprop-2-enoate (I) Crystal data

**Table:**

C_15_H_21_N_2_O_2_^+^·C_4_H_3_O_4_^−^	*F*(000) = 400
*M**_r_* = 376.40	*D*_x_ = 1.279 Mg m^−^^3^
Monoclinic, *P*2_1_	Mo *K*α radiation, λ = 0.71073 Å
*a* = 7.9555 (4) Å	Cell parameters from 9372 reflections
*b* = 13.3696 (7) Å	θ = 2.7–25.4°
*c* = 9.9708 (5) Å	µ = 0.10 mm^−^^1^
β = 112.874 (2)°	*T* = 200 K
*V* = 977.12 (9) Å^3^	Block, colorless
*Z* = 2	0.24 × 0.2 × 0.2 mm

### [2-(4-Acetyloxy-1H-indol-3-yl)ethyl](methyl)ethylazanium 3-carboxyprop-2-enoate (I) Data collection

**Table:**

Bruker D8 Venture CMOS diffractometer	3232 reflections with *I* > 2σ(*I*)
φ and ω scans	*R*_int_ = 0.036
Absorption correction: multi-scan (SADABS; Bruker, 2018)	θ_max_ = 25.4°, θ_min_ = 2.8°
*T*_min_ = 0.708, *T*_max_ = 0.745	*h* = −9→9
21973 measured reflections	*k* = −16→16
3536 independent reflections	*l* = −11→12

### [2-(4-Acetyloxy-1H-indol-3-yl)ethyl](methyl)ethylazanium 3-carboxyprop-2-enoate (I) Refinement

**Table:**

Refinement on *F*^2^	Hydrogen site location: mixed
Least-squares matrix: full	H atoms treated by a mixture of independent and constrained refinement
*R*[*F*^2^ > 2σ(*F*^2^)] = 0.052	*w* = 1/[σ^2^(*F*_o_^2^) + (0.0859*P*)^2^ + 0.4056*P*] where *P* = (*F*_o_^2^ + 2*F*_c_^2^)/3
*wR*(*F*^2^) = 0.146	(Δ/σ)_max_ < 0.001
*S* = 1.04	Δρ_max_ = 0.39 e Å^−^^3^
3536 reflections	Δρ_min_ = −0.55 e Å^−^^3^
271 parameters	Absolute structure: Flack *x* determined using 1411 quotients [(*I*^+^)-(*I*^-^)]/[(*I*^+^)+(*I*^-^)] (Parsons *et al.*, 2013)
15 restraints	Absolute structure parameter: −0.3 (3)

### [2-(4-Acetyloxy-1H-indol-3-yl)ethyl](methyl)ethylazanium 3-carboxyprop-2-enoate (I) Special details

**Table:**

Geometry. All esds (except the esd in the dihedral angle between two l.s. planes) are estimated using the full covariance matrix. The cell esds are taken into account individually in the estimation of esds in distances, angles and torsion angles; correlations between esds in cell parameters are only used when they are defined by crystal symmetry. An approximate (isotropic) treatment of cell esds is used for estimating esds involving l.s. planes.

### [2-(4-Acetyloxy-1H-indol-3-yl)ethyl](methyl)ethylazanium 3-carboxyprop-2-enoate (I) Fractional atomic coordinates and isotropic or equivalent isotropic displacement parameters (Å^2^)

**Table:**

	*x*	*y*	*z*	*U*_iso_*/*U*_eq_	Occ. (<1)
O1	0.8969 (4)	0.6685 (2)	0.3476 (3)	0.0419 (7)	
O2	0.6590 (4)	0.7649 (2)	0.2143 (3)	0.0527 (8)	
O3	0.6091 (4)	0.4634 (4)	0.5977 (3)	0.0643 (11)	
O4	0.3948 (4)	0.4494 (4)	0.3791 (3)	0.0726 (12)	
O5	−0.1636 (4)	0.4458 (3)	0.4883 (3)	0.0464 (7)	
H5A	−0.228 (7)	0.461 (5)	0.541 (5)	0.070*	
O6	0.0341 (4)	0.5232 (3)	0.6851 (3)	0.0530 (8)	
N1	0.8829 (5)	0.5556 (3)	−0.1012 (4)	0.0445 (8)	
H1	0.909 (7)	0.546 (4)	−0.177 (3)	0.053*	
C1	0.6770 (8)	0.7029 (4)	0.4462 (5)	0.0609 (13)	
H1A	0.771261	0.727703	0.536780	0.091*	
H1B	0.656163	0.631605	0.456386	0.091*	
H1C	0.563452	0.739969	0.425935	0.091*	
C2	0.7388 (6)	0.7176 (3)	0.3229 (4)	0.0398 (9)	
C3	0.9685 (5)	0.6723 (3)	0.2378 (4)	0.0377 (9)	
C4	1.1178 (6)	0.7317 (3)	0.2611 (5)	0.0505 (11)	
H4	1.165836	0.772222	0.345887	0.061*	
C5	1.1983 (7)	0.7322 (4)	0.1596 (6)	0.0581 (13)	
H5	1.303718	0.772121	0.177668	0.070*	
C6	1.1292 (7)	0.6765 (4)	0.0340 (6)	0.0547 (12)	
H6	1.183090	0.678752	−0.035920	0.066*	
C7	0.9784 (6)	0.6171 (3)	0.0127 (5)	0.0399 (9)	
C8	0.8952 (5)	0.6117 (3)	0.1155 (4)	0.0344 (8)	
C9	0.7462 (5)	0.5416 (3)	0.0580 (4)	0.0347 (8)	
C10	0.7465 (6)	0.5098 (3)	−0.0720 (4)	0.0413 (9)	
H10	0.663545	0.462369	−0.133842	0.050*	
C11	0.6170 (6)	0.5075 (3)	0.1251 (5)	0.0462 (10)	
H11A	0.500209	0.544176	0.080710	0.055*	0.760 (7)
H11B	0.669365	0.522179	0.230754	0.055*	0.760 (7)
H11C	0.493724	0.534844	0.069534	0.055*	0.240 (7)
H11D	0.658515	0.532211	0.226482	0.055*	0.240 (7)
N2	0.4319 (8)	0.3670 (4)	0.1512 (6)	0.0478 (14)	0.760 (7)
H2	0.460 (10)	0.389 (6)	0.240 (4)	0.057*	0.760 (7)
C12	0.583 (2)	0.3971 (6)	0.1019 (15)	0.0747 (14)	0.760 (7)
H12A	0.695565	0.359695	0.158198	0.090*	0.760 (7)
H12B	0.546626	0.380879	−0.002452	0.090*	0.760 (7)
C13	0.4415 (12)	0.2578 (5)	0.1741 (9)	0.0747 (14)	0.760 (7)
H13A	0.434068	0.222514	0.084645	0.090*	0.760 (7)
H13B	0.339870	0.234739	0.200220	0.090*	0.760 (7)
C14	0.6257 (12)	0.2371 (6)	0.2991 (9)	0.0747 (14)	0.760 (7)
H14A	0.609720	0.186452	0.364171	0.112*	0.760 (7)
H14B	0.671948	0.299017	0.353700	0.112*	0.760 (7)
H14C	0.712937	0.212798	0.259233	0.112*	0.760 (7)
C15	0.2372 (8)	0.3878 (5)	0.0459 (7)	0.0518 (14)	0.760 (7)
H15A	0.222136	0.459583	0.024871	0.078*	0.760 (7)
H15B	0.152161	0.366435	0.089854	0.078*	0.760 (7)
H15C	0.211710	0.350719	−0.044649	0.078*	0.760 (7)
N2A	0.5331 (17)	0.3315 (9)	0.2166 (13)	0.024 (3)	0.240 (7)
H2A	0.51 (2)	0.371 (11)	0.278 (13)	0.029*	0.240 (7)
C12A	0.609 (7)	0.3915 (11)	0.124 (5)	0.0747 (14)	0.240 (7)
H12C	0.539526	0.371383	0.021726	0.090*	0.240 (7)
H12D	0.736058	0.367924	0.149239	0.090*	0.240 (7)
C13A	0.555 (4)	0.2363 (15)	0.305 (2)	0.0747 (14)	0.240 (7)
H13C	0.640638	0.192254	0.282152	0.090*	0.240 (7)
H13D	0.435712	0.202054	0.269481	0.090*	0.240 (7)
C14A	0.623 (4)	0.242 (2)	0.471 (2)	0.0747 (14)	0.240 (7)
H14D	0.724365	0.195394	0.515462	0.112*	0.240 (7)
H14E	0.523313	0.224946	0.501539	0.112*	0.240 (7)
H14F	0.664930	0.310475	0.503391	0.112*	0.240 (7)
C15A	0.335 (2)	0.3065 (18)	0.116 (2)	0.0518 (14)	0.240 (7)
H15D	0.277454	0.268541	0.170713	0.078*	0.240 (7)
H15E	0.334919	0.266491	0.033717	0.078*	0.240 (7)
H15F	0.267400	0.368689	0.079997	0.078*	0.240 (7)
C16	0.4441 (5)	0.4620 (3)	0.5089 (4)	0.0363 (8)	
C17	0.3065 (5)	0.4772 (3)	0.5751 (4)	0.0384 (9)	
H17	0.348389	0.491198	0.676267	0.046*	
C18	0.1302 (5)	0.4720 (3)	0.4987 (4)	0.0375 (8)	
H18	0.087892	0.460653	0.396827	0.045*	
C19	−0.0063 (5)	0.4833 (3)	0.5668 (4)	0.0366 (9)	

### [2-(4-Acetyloxy-1H-indol-3-yl)ethyl](methyl)ethylazanium 3-carboxyprop-2-enoate (I) Atomic displacement parameters (Å^2^)

**Table:**

	*U*^11^	*U*^22^	*U*^33^	*U*^12^	*U*^13^	*U*^23^
O1	0.0513 (17)	0.0443 (15)	0.0315 (13)	0.0013 (13)	0.0174 (12)	−0.0005 (12)
O2	0.0541 (18)	0.060 (2)	0.0505 (18)	0.0116 (16)	0.0277 (15)	0.0128 (16)
O3	0.0266 (14)	0.130 (3)	0.0416 (16)	0.0008 (18)	0.0187 (12)	−0.0061 (19)
O4	0.0403 (16)	0.144 (4)	0.0440 (18)	0.002 (2)	0.0277 (14)	−0.027 (2)
O5	0.0324 (14)	0.069 (2)	0.0456 (16)	−0.0014 (13)	0.0234 (12)	−0.0050 (14)
O6	0.0393 (15)	0.087 (2)	0.0433 (16)	−0.0022 (15)	0.0274 (13)	−0.0111 (16)
N1	0.057 (2)	0.051 (2)	0.0400 (18)	0.0093 (17)	0.0352 (17)	0.0022 (15)
C1	0.089 (4)	0.057 (3)	0.059 (3)	0.012 (3)	0.054 (3)	0.006 (2)
C2	0.052 (2)	0.036 (2)	0.039 (2)	−0.0023 (17)	0.0260 (18)	−0.0033 (17)
C3	0.038 (2)	0.0386 (19)	0.039 (2)	0.0028 (16)	0.0168 (17)	0.0023 (16)
C4	0.043 (2)	0.051 (3)	0.051 (2)	−0.0071 (19)	0.0112 (19)	0.000 (2)
C5	0.043 (2)	0.063 (3)	0.073 (3)	−0.009 (2)	0.028 (2)	0.005 (3)
C6	0.049 (3)	0.056 (3)	0.077 (3)	0.003 (2)	0.045 (2)	0.012 (2)
C7	0.040 (2)	0.042 (2)	0.046 (2)	0.0097 (17)	0.0263 (18)	0.0063 (17)
C8	0.0338 (19)	0.0388 (19)	0.0367 (19)	0.0029 (15)	0.0201 (16)	0.0036 (15)
C9	0.0394 (19)	0.0374 (19)	0.0324 (18)	0.0029 (15)	0.0194 (16)	−0.0015 (15)
C10	0.045 (2)	0.047 (2)	0.0367 (19)	0.0011 (18)	0.0205 (17)	−0.0045 (17)
C11	0.052 (2)	0.053 (2)	0.048 (2)	−0.0120 (19)	0.035 (2)	−0.0122 (19)
N2	0.055 (3)	0.046 (3)	0.054 (3)	−0.016 (3)	0.034 (3)	−0.018 (3)
C12	0.100 (4)	0.061 (2)	0.084 (3)	−0.020 (2)	0.060 (3)	−0.009 (2)
C13	0.100 (4)	0.061 (2)	0.084 (3)	−0.020 (2)	0.060 (3)	−0.009 (2)
C14	0.100 (4)	0.061 (2)	0.084 (3)	−0.020 (2)	0.060 (3)	−0.009 (2)
C15	0.043 (3)	0.064 (4)	0.046 (3)	0.000 (3)	0.014 (2)	−0.016 (3)
N2A	0.024 (6)	0.025 (6)	0.021 (6)	−0.009 (5)	0.007 (5)	0.004 (5)
C12A	0.100 (4)	0.061 (2)	0.084 (3)	−0.020 (2)	0.060 (3)	−0.009 (2)
C13A	0.100 (4)	0.061 (2)	0.084 (3)	−0.020 (2)	0.060 (3)	−0.009 (2)
C14A	0.100 (4)	0.061 (2)	0.084 (3)	−0.020 (2)	0.060 (3)	−0.009 (2)
C15A	0.043 (3)	0.064 (4)	0.046 (3)	0.000 (3)	0.014 (2)	−0.016 (3)
C16	0.0355 (18)	0.045 (2)	0.0360 (19)	−0.0002 (17)	0.0220 (15)	−0.0020 (16)
C17	0.0327 (18)	0.056 (2)	0.0319 (17)	0.0024 (17)	0.0184 (15)	0.0001 (17)
C18	0.0340 (18)	0.051 (2)	0.0343 (18)	0.0040 (17)	0.0209 (15)	0.0009 (17)
C19	0.0316 (18)	0.048 (2)	0.0373 (19)	0.0052 (16)	0.0209 (15)	0.0039 (17)

### [2-(4-Acetyloxy-1H-indol-3-yl)ethyl](methyl)ethylazanium 3-carboxyprop-2-enoate (I) Geometric parameters (Å, º)

**Table:**

O1—C2	1.354 (5)	N2—C12	1.517 (10)
O1—C3	1.418 (5)	N2—C13	1.476 (8)
O2—C2	1.200 (5)	N2—C15	1.521 (8)
O3—C16	1.267 (5)	C12—H12A	0.9900
O4—C16	1.209 (5)	C12—H12B	0.9900
O5—H5A	0.886 (15)	C13—H13A	0.9900
O5—C19	1.292 (5)	C13—H13B	0.9900
O6—C19	1.218 (5)	C13—C14	1.535 (10)
N1—H1	0.862 (14)	C14—H14A	0.9800
N1—C7	1.368 (6)	C14—H14B	0.9800
N1—C10	1.372 (6)	C14—H14C	0.9800
C1—H1A	0.9800	C15—H15A	0.9800
C1—H1B	0.9800	C15—H15B	0.9800
C1—H1C	0.9800	C15—H15C	0.9800
C1—C2	1.504 (6)	N2A—H2A	0.870 (15)
C3—C4	1.371 (6)	N2A—C12A	1.518 (14)
C3—C8	1.390 (6)	N2A—C13A	1.521 (13)
C4—H4	0.9500	N2A—C15A	1.538 (12)
C4—C5	1.392 (7)	C12A—H12C	0.9900
C5—H5	0.9500	C12A—H12D	0.9900
C5—C6	1.376 (8)	C13A—H13C	0.9900
C6—H6	0.9500	C13A—H13D	0.9900
C6—C7	1.385 (7)	C13A—C14A	1.532 (14)
C7—C8	1.422 (5)	C14A—H14D	0.9800
C8—C9	1.443 (6)	C14A—H14E	0.9800
C9—C10	1.365 (5)	C14A—H14F	0.9800
C9—C11	1.500 (5)	C15A—H15D	0.9800
C10—H10	0.9500	C15A—H15E	0.9800
C11—H11A	0.9900	C15A—H15F	0.9800
C11—H11B	0.9900	C16—C17	1.495 (5)
C11—H11C	0.9900	C17—H17	0.9500
C11—H11D	0.9900	C17—C18	1.312 (5)
C11—C12	1.501 (9)	C18—H18	0.9500
C11—C12A	1.551 (14)	C18—C19	1.496 (5)
N2—H2	0.875 (15)		
			
C2—O1—C3	117.8 (3)	H12A—C12—H12B	108.3
C19—O5—H5A	101 (4)	N2—C13—H13A	110.5
C7—N1—H1	125 (3)	N2—C13—H13B	110.5
C7—N1—C10	108.7 (3)	N2—C13—C14	106.2 (6)
C10—N1—H1	126 (3)	H13A—C13—H13B	108.7
H1A—C1—H1B	109.5	C14—C13—H13A	110.5
H1A—C1—H1C	109.5	C14—C13—H13B	110.5
H1B—C1—H1C	109.5	C13—C14—H14A	109.5
C2—C1—H1A	109.5	C13—C14—H14B	109.5
C2—C1—H1B	109.5	C13—C14—H14C	109.5
C2—C1—H1C	109.5	H14A—C14—H14B	109.5
O1—C2—C1	111.3 (4)	H14A—C14—H14C	109.5
O2—C2—O1	123.2 (4)	H14B—C14—H14C	109.5
O2—C2—C1	125.4 (4)	N2—C15—H15A	109.5
C4—C3—O1	118.1 (4)	N2—C15—H15B	109.5
C4—C3—C8	121.8 (4)	N2—C15—H15C	109.5
C8—C3—O1	119.9 (3)	H15A—C15—H15B	109.5
C3—C4—H4	120.2	H15A—C15—H15C	109.5
C3—C4—C5	119.5 (4)	H15B—C15—H15C	109.5
C5—C4—H4	120.2	C12A—N2A—H2A	109 (10)
C4—C5—H5	119.2	C12A—N2A—C13A	144 (2)
C6—C5—C4	121.7 (4)	C12A—N2A—C15A	106 (2)
C6—C5—H5	119.2	C13A—N2A—H2A	97 (10)
C5—C6—H6	121.1	C13A—N2A—C15A	93.2 (16)
C5—C6—C7	117.8 (4)	C15A—N2A—H2A	101 (10)
C7—C6—H6	121.1	C11—C12A—H12C	106.5
N1—C7—C6	129.8 (4)	C11—C12A—H12D	106.5
N1—C7—C8	107.7 (3)	N2A—C12A—C11	123.2 (16)
C6—C7—C8	122.5 (4)	N2A—C12A—H12C	106.5
C3—C8—C7	116.7 (4)	N2A—C12A—H12D	106.5
C3—C8—C9	136.6 (4)	H12C—C12A—H12D	106.5
C7—C8—C9	106.7 (3)	N2A—C13A—H13C	107.4
C8—C9—C11	128.3 (3)	N2A—C13A—H13D	107.4
C10—C9—C8	105.9 (3)	N2A—C13A—C14A	119.8 (18)
C10—C9—C11	125.7 (4)	H13C—C13A—H13D	106.9
N1—C10—H10	124.6	C14A—C13A—H13C	107.4
C9—C10—N1	110.9 (4)	C14A—C13A—H13D	107.4
C9—C10—H10	124.6	C13A—C14A—H14D	109.5
C9—C11—H11A	109.6	C13A—C14A—H14E	109.5
C9—C11—H11B	109.6	C13A—C14A—H14F	109.5
C9—C11—H11C	109.8	H14D—C14A—H14E	109.5
C9—C11—H11D	109.8	H14D—C14A—H14F	109.5
C9—C11—C12	110.2 (5)	H14E—C14A—H14F	109.5
C9—C11—C12A	109.4 (10)	N2A—C15A—H15D	109.5
H11A—C11—H11B	108.1	N2A—C15A—H15E	109.5
H11C—C11—H11D	108.2	N2A—C15A—H15F	109.5
C12—C11—H11A	109.6	H15D—C15A—H15E	109.5
C12—C11—H11B	109.6	H15D—C15A—H15F	109.5
C12A—C11—H11C	109.8	H15E—C15A—H15F	109.5
C12A—C11—H11D	109.8	O3—C16—C17	115.3 (3)
C12—N2—H2	108 (6)	O4—C16—O3	124.5 (3)
C12—N2—C15	116.7 (8)	O4—C16—C17	120.2 (3)
C13—N2—H2	101 (6)	C16—C17—H17	118.7
C13—N2—C12	108.3 (6)	C18—C17—C16	122.5 (3)
C13—N2—C15	105.4 (5)	C18—C17—H17	118.7
C15—N2—H2	115 (5)	C17—C18—H18	118.9
C11—C12—N2	109.3 (6)	C17—C18—C19	122.2 (3)
C11—C12—H12A	109.8	C19—C18—H18	118.9
C11—C12—H12B	109.8	O5—C19—C18	112.9 (3)
N2—C12—H12A	109.8	O6—C19—O5	125.9 (3)
N2—C12—H12B	109.8	O6—C19—C18	121.2 (3)
			
O1—C3—C4—C5	176.0 (4)	C7—C8—C9—C11	178.7 (4)
O1—C3—C8—C7	−177.9 (3)	C8—C3—C4—C5	0.4 (6)
O1—C3—C8—C9	3.9 (7)	C8—C9—C10—N1	−0.8 (5)
O3—C16—C17—C18	−175.9 (4)	C8—C9—C11—C12	−139.1 (7)
O4—C16—C17—C18	3.9 (7)	C8—C9—C11—C12A	−129 (2)
N1—C7—C8—C3	−177.6 (3)	C9—C11—C12—N2	−172.0 (7)
N1—C7—C8—C9	1.1 (4)	C9—C11—C12A—N2A	164 (3)
C2—O1—C3—C4	105.8 (4)	C10—N1—C7—C6	178.4 (4)
C2—O1—C3—C8	−78.5 (5)	C10—N1—C7—C8	−1.6 (5)
C3—O1—C2—O2	−1.2 (6)	C10—C9—C11—C12	39.6 (9)
C3—O1—C2—C1	177.7 (4)	C10—C9—C11—C12A	49 (2)
C3—C4—C5—C6	1.8 (7)	C11—C9—C10—N1	−179.8 (4)
C3—C8—C9—C10	178.2 (5)	C12—N2—C13—C14	63.8 (9)
C3—C8—C9—C11	−2.9 (7)	C13—N2—C12—C11	−161.4 (8)
C4—C3—C8—C7	−2.3 (6)	C15—N2—C12—C11	80.0 (10)
C4—C3—C8—C9	179.4 (4)	C15—N2—C13—C14	−170.6 (5)
C4—C5—C6—C7	−1.7 (8)	C12A—N2A—C13A—C14A	110 (4)
C5—C6—C7—N1	179.6 (4)	C13A—N2A—C12A—C11	−144 (3)
C5—C6—C7—C8	−0.4 (7)	C15A—N2A—C12A—C11	97 (4)
C6—C7—C8—C3	2.4 (6)	C15A—N2A—C13A—C14A	−128 (2)
C6—C7—C8—C9	−178.9 (4)	C16—C17—C18—C19	177.5 (4)
C7—N1—C10—C9	1.6 (5)	C17—C18—C19—O5	−159.5 (4)
C7—C8—C9—C10	−0.2 (4)	C17—C18—C19—O6	19.7 (6)

### [2-(4-Acetyloxy-1H-indol-3-yl)ethyl](methyl)ethylazanium 3-carboxyprop-2-enoate (I) Hydrogen-bond geometry (Å, º)

**Table:**

*D*—H···*A*	*D*—H	H···*A*	*D*···*A*	*D*—H···*A*
N1—H1···O6^i^	0.86 (1)	2.02 (2)	2.858 (4)	165 (5)
N2—H2···O4	0.88 (2)	1.85 (4)	2.644 (6)	150 (7)
N2*A*—H2*A*···O4	0.87 (2)	1.94 (6)	2.776 (14)	162 (17)
O5—H5*A*···O3^ii^	0.89 (2)	1.61 (2)	2.459 (4)	160 (6)

Symmetry codes: (i) *x*+1, *y*, *z*−1; (ii) *x*−1, *y*, *z*.

### [2-(4-Acetyloxy-1H-indol-3-yl)ethyl](methyl)prop-2-enylazanium 3-carboxyprop-2-enoate (II) Crystal data

**Table:**

C_16_H_21_N_2_O_2_^+^·C_4_H_3_O_4_^−^	*F*(000) = 412
*M**_r_* = 388.41	*D*_x_ = 1.269 Mg m^−^^3^
Monoclinic, *P*2_1_	Mo *K*α radiation, λ = 0.71073 Å
*a* = 7.9702 (4) Å	Cell parameters from 9882 reflections
*b* = 14.1788 (7) Å	θ = 2.7–25.6°
*c* = 9.8035 (5) Å	µ = 0.09 mm^−^^1^
β = 113.394 (2)°	*T* = 297 K
*V* = 1016.80 (9) Å^3^	BLOCK, colourless
*Z* = 2	0.34 × 0.24 × 0.2 mm

### [2-(4-Acetyloxy-1H-indol-3-yl)ethyl](methyl)prop-2-enylazanium 3-carboxyprop-2-enoate (II) Data collection

**Table:**

Bruker D8 Venture CMOS diffractometer	3516 reflections with *I* > 2σ(*I*)
φ and ω scans	*R*_int_ = 0.039
Absorption correction: multi-scan (SADABS; Bruker, 2018)	θ_max_ = 25.7°, θ_min_ = 2.8°
*T*_min_ = 0.686, *T*_max_ = 0.745	*h* = −9→9
26393 measured reflections	*k* = −17→17
3797 independent reflections	*l* = −11→11

### [2-(4-Acetyloxy-1H-indol-3-yl)ethyl](methyl)prop-2-enylazanium 3-carboxyprop-2-enoate (II) Refinement

**Table:**

Refinement on *F*^2^	Hydrogen site location: mixed
Least-squares matrix: full	H atoms treated by a mixture of independent and constrained refinement
*R*[*F*^2^ > 2σ(*F*^2^)] = 0.043	*w* = 1/[σ^2^(*F*_o_^2^) + (0.0592*P*)^2^ + 0.2448*P*] where *P* = (*F*_o_^2^ + 2*F*_c_^2^)/3
*wR*(*F*^2^) = 0.113	(Δ/σ)_max_ < 0.001
*S* = 1.04	Δρ_max_ = 0.27 e Å^−^^3^
3797 reflections	Δρ_min_ = −0.16 e Å^−^^3^
264 parameters	Absolute structure: Flack *x* determined using 1577 quotients [(*I*^+^)-(*I*^-^)]/[(*I*^+^)+(*I*^-^)] (Parsons *et al.*, 2013)
4 restraints	Absolute structure parameter: 0.4 (3)

### [2-(4-Acetyloxy-1H-indol-3-yl)ethyl](methyl)prop-2-enylazanium 3-carboxyprop-2-enoate (II) Special details

**Table:**

Geometry. All esds (except the esd in the dihedral angle between two l.s. planes) are estimated using the full covariance matrix. The cell esds are taken into account individually in the estimation of esds in distances, angles and torsion angles; correlations between esds in cell parameters are only used when they are defined by crystal symmetry. An approximate (isotropic) treatment of cell esds is used for estimating esds involving l.s. planes.

### [2-(4-Acetyloxy-1H-indol-3-yl)ethyl](methyl)prop-2-enylazanium 3-carboxyprop-2-enoate (II) Fractional atomic coordinates and isotropic or equivalent isotropic displacement parameters (Å^2^)

**Table:**

	*x*	*y*	*z*	*U*_iso_*/*U*_eq_	
O1	0.0798 (3)	0.7896 (2)	0.2116 (3)	0.0688 (8)	
O2	−0.0241 (3)	0.69624 (16)	0.3445 (2)	0.0496 (5)	
O3	0.5149 (3)	0.4747 (3)	0.3619 (3)	0.0834 (11)	
O4	0.5188 (3)	0.4841 (3)	0.5856 (3)	0.0701 (8)	
H4A	0.397 (2)	0.484 (4)	0.543 (5)	0.105*	
O5	1.1781 (3)	0.53485 (19)	0.6822 (2)	0.0559 (6)	
O6	1.1837 (2)	0.47635 (18)	0.4735 (2)	0.0493 (5)	
N1	−0.4734 (4)	0.5921 (2)	−0.1031 (3)	0.0585 (8)	
H1	−0.577 (3)	0.580 (3)	−0.176 (3)	0.070*	
N2	0.2259 (4)	0.4086 (2)	0.1361 (3)	0.0499 (6)	
H2	0.297 (4)	0.436 (3)	0.221 (3)	0.060*	
C1	0.2902 (5)	0.7280 (3)	0.4444 (5)	0.0754 (12)	
H1A	0.377223	0.771990	0.436158	0.113*	
H1B	0.330726	0.664822	0.439678	0.113*	
H1C	0.279217	0.737271	0.537570	0.113*	
C2	0.1079 (5)	0.7436 (2)	0.3193 (4)	0.0506 (8)	
C3	−0.2038 (4)	0.7039 (2)	0.2363 (3)	0.0464 (7)	
C4	−0.3218 (6)	0.7650 (3)	0.2617 (5)	0.0642 (9)	
H4	−0.281087	0.803090	0.345811	0.077*	
C5	−0.5046 (6)	0.7694 (3)	0.1595 (6)	0.0776 (12)	
H5	−0.584090	0.810842	0.177293	0.093*	
C6	−0.5689 (5)	0.7147 (3)	0.0352 (5)	0.0685 (11)	
H6	−0.690394	0.718337	−0.031497	0.082*	
C7	−0.4473 (4)	0.6531 (2)	0.0109 (4)	0.0494 (8)	
C8	−0.2618 (4)	0.6455 (2)	0.1114 (3)	0.0401 (6)	
C9	−0.1793 (4)	0.5762 (2)	0.0515 (3)	0.0418 (6)	
C10	−0.3140 (5)	0.5459 (3)	−0.0778 (3)	0.0531 (8)	
H10	−0.299037	0.500150	−0.139982	0.064*	
C11	0.0171 (4)	0.5439 (2)	0.1121 (4)	0.0491 (7)	
H11A	0.082400	0.578146	0.062769	0.059*	
H11B	0.074142	0.558138	0.217389	0.059*	
C12	0.0315 (4)	0.4409 (2)	0.0897 (4)	0.0554 (8)	
H12A	−0.036085	0.425881	−0.014403	0.066*	
H12B	−0.024264	0.406630	0.146635	0.066*	
C13	0.2451 (6)	0.3047 (3)	0.1669 (5)	0.0656 (10)	
H13A	0.165728	0.271014	0.078805	0.079*	
H13B	0.369974	0.285978	0.188000	0.079*	
C14	0.1994 (6)	0.2777 (3)	0.2918 (5)	0.0745 (11)	
H14	0.260747	0.308976	0.381312	0.089*	
C15	0.0818 (9)	0.2144 (4)	0.2897 (8)	0.1105 (19)	
H15A	0.017104	0.181308	0.202624	0.133*	
H15B	0.061757	0.201983	0.375131	0.133*	
C16	0.3019 (6)	0.4354 (4)	0.0257 (4)	0.0741 (12)	
H16A	0.425726	0.413518	0.058781	0.111*	
H16B	0.299241	0.502755	0.015387	0.111*	
H16C	0.229752	0.407199	−0.068548	0.111*	
C17	0.5946 (3)	0.4843 (2)	0.4951 (3)	0.0412 (6)	
C18	0.7971 (3)	0.4950 (2)	0.5624 (3)	0.0418 (6)	
H18	0.853754	0.504450	0.664454	0.050*	
C19	0.8996 (3)	0.4919 (2)	0.4870 (3)	0.0406 (6)	
H19	0.844150	0.482380	0.384840	0.049*	
C20	1.1021 (3)	0.5030 (2)	0.5574 (3)	0.0372 (6)	

### [2-(4-Acetyloxy-1H-indol-3-yl)ethyl](methyl)prop-2-enylazanium 3-carboxyprop-2-enoate (II) Atomic displacement parameters (Å^2^)

**Table:**

	*U*^11^	*U*^22^	*U*^33^	*U*^12^	*U*^13^	*U*^23^
O1	0.0517 (14)	0.0828 (19)	0.0643 (16)	−0.0064 (13)	0.0149 (12)	0.0250 (14)
O2	0.0521 (12)	0.0543 (12)	0.0358 (10)	−0.0098 (10)	0.0102 (9)	−0.0002 (9)
O3	0.0276 (10)	0.176 (3)	0.0441 (13)	−0.0232 (16)	0.0119 (9)	−0.0257 (17)
O4	0.0255 (10)	0.142 (3)	0.0462 (12)	−0.0035 (14)	0.0177 (9)	−0.0141 (15)
O5	0.0243 (9)	0.0955 (18)	0.0443 (12)	−0.0058 (10)	0.0098 (9)	−0.0179 (11)
O6	0.0274 (9)	0.0772 (15)	0.0464 (11)	−0.0007 (10)	0.0177 (8)	−0.0114 (10)
N1	0.0341 (14)	0.076 (2)	0.0502 (15)	−0.0136 (13)	0.0009 (12)	0.0058 (14)
N2	0.0438 (14)	0.0591 (16)	0.0439 (14)	−0.0010 (12)	0.0144 (11)	−0.0097 (12)
C1	0.053 (2)	0.077 (3)	0.071 (2)	−0.0121 (19)	−0.0019 (18)	0.006 (2)
C2	0.0490 (17)	0.0470 (17)	0.0483 (18)	−0.0062 (14)	0.0113 (14)	−0.0035 (14)
C3	0.0439 (16)	0.0499 (17)	0.0452 (16)	−0.0041 (13)	0.0176 (13)	0.0034 (14)
C4	0.071 (2)	0.063 (2)	0.069 (2)	0.0029 (18)	0.039 (2)	−0.0023 (18)
C5	0.064 (2)	0.076 (3)	0.110 (4)	0.019 (2)	0.052 (3)	0.016 (3)
C6	0.0366 (17)	0.080 (3)	0.089 (3)	0.0054 (17)	0.0247 (18)	0.019 (2)
C7	0.0334 (14)	0.0576 (18)	0.0536 (18)	−0.0082 (13)	0.0134 (13)	0.0112 (15)
C8	0.0327 (13)	0.0462 (15)	0.0385 (14)	−0.0042 (11)	0.0110 (11)	0.0070 (12)
C9	0.0397 (14)	0.0479 (15)	0.0325 (13)	−0.0044 (12)	0.0087 (11)	0.0030 (12)
C10	0.0490 (17)	0.064 (2)	0.0374 (15)	−0.0099 (16)	0.0078 (13)	−0.0025 (14)
C11	0.0408 (15)	0.0561 (18)	0.0430 (15)	0.0002 (14)	0.0087 (13)	−0.0020 (14)
C12	0.0429 (17)	0.0551 (19)	0.063 (2)	−0.0037 (14)	0.0151 (16)	−0.0039 (15)
C13	0.062 (2)	0.062 (2)	0.065 (2)	0.0093 (17)	0.0170 (18)	−0.0060 (17)
C14	0.089 (3)	0.056 (2)	0.078 (3)	0.006 (2)	0.033 (2)	0.003 (2)
C15	0.117 (5)	0.094 (4)	0.118 (4)	−0.012 (3)	0.044 (4)	0.018 (4)
C16	0.071 (2)	0.105 (3)	0.058 (2)	−0.005 (2)	0.039 (2)	−0.006 (2)
C17	0.0252 (12)	0.0562 (17)	0.0398 (15)	−0.0030 (13)	0.0104 (11)	−0.0064 (13)
C18	0.0264 (12)	0.0607 (18)	0.0370 (14)	−0.0035 (12)	0.0113 (11)	−0.0052 (13)
C19	0.0233 (12)	0.0588 (17)	0.0370 (13)	−0.0041 (12)	0.0091 (10)	−0.0037 (12)
C20	0.0232 (11)	0.0485 (15)	0.0384 (14)	−0.0016 (11)	0.0107 (10)	−0.0002 (12)

### [2-(4-Acetyloxy-1H-indol-3-yl)ethyl](methyl)prop-2-enylazanium 3-carboxyprop-2-enoate (II) Geometric parameters (Å, º)

**Table:**

O1—C2	1.185 (4)	C6—C7	1.394 (6)
O2—C2	1.351 (4)	C7—C8	1.419 (4)
O2—C3	1.408 (4)	C8—C9	1.431 (5)
O3—C17	1.212 (3)	C9—C10	1.365 (4)
O4—H4A	0.894 (14)	C9—C11	1.508 (4)
O4—C17	1.256 (3)	C10—H10	0.9300
O5—C20	1.216 (3)	C11—H11A	0.9700
O6—C20	1.290 (3)	C11—H11B	0.9700
N1—H1	0.867 (14)	C11—C12	1.488 (5)
N1—C7	1.362 (5)	C12—H12A	0.9700
N1—C10	1.362 (5)	C12—H12B	0.9700
N2—H2	0.890 (14)	C13—H13A	0.9700
N2—C12	1.502 (4)	C13—H13B	0.9700
N2—C13	1.499 (5)	C13—C14	1.460 (6)
N2—C16	1.484 (4)	C14—H14	0.9300
C1—H1A	0.9600	C14—C15	1.292 (7)
C1—H1B	0.9600	C15—H15A	0.9300
C1—H1C	0.9600	C15—H15B	0.9300
C1—C2	1.499 (5)	C16—H16A	0.9600
C3—C4	1.372 (5)	C16—H16B	0.9600
C3—C8	1.396 (4)	C16—H16C	0.9600
C4—H4	0.9300	C17—C18	1.489 (3)
C4—C5	1.404 (6)	C18—H18	0.9300
C5—H5	0.9300	C18—C19	1.302 (4)
C5—C6	1.361 (7)	C19—H19	0.9300
C6—H6	0.9300	C19—C20	1.491 (3)
			
C2—O2—C3	117.2 (2)	C9—C10—H10	124.7
C17—O4—H4A	114 (3)	C9—C11—H11A	109.3
C7—N1—H1	126 (3)	C9—C11—H11B	109.3
C7—N1—C10	109.2 (3)	H11A—C11—H11B	107.9
C10—N1—H1	124 (3)	C12—C11—C9	111.7 (3)
C12—N2—H2	110 (3)	C12—C11—H11A	109.3
C13—N2—H2	105 (3)	C12—C11—H11B	109.3
C13—N2—C12	111.8 (3)	N2—C12—H12A	109.1
C16—N2—H2	106 (3)	N2—C12—H12B	109.1
C16—N2—C12	111.8 (3)	C11—C12—N2	112.7 (3)
C16—N2—C13	111.1 (3)	C11—C12—H12A	109.1
H1A—C1—H1B	109.5	C11—C12—H12B	109.1
H1A—C1—H1C	109.5	H12A—C12—H12B	107.8
H1B—C1—H1C	109.5	N2—C13—H13A	109.0
C2—C1—H1A	109.5	N2—C13—H13B	109.0
C2—C1—H1B	109.5	H13A—C13—H13B	107.8
C2—C1—H1C	109.5	C14—C13—N2	112.8 (3)
O1—C2—O2	123.5 (3)	C14—C13—H13A	109.0
O1—C2—C1	126.1 (3)	C14—C13—H13B	109.0
O2—C2—C1	110.4 (3)	C13—C14—H14	116.9
C4—C3—O2	118.5 (3)	C15—C14—C13	126.1 (5)
C4—C3—C8	121.4 (3)	C15—C14—H14	116.9
C8—C3—O2	119.9 (3)	C14—C15—H15A	120.0
C3—C4—H4	120.3	C14—C15—H15B	120.0
C3—C4—C5	119.4 (4)	H15A—C15—H15B	120.0
C5—C4—H4	120.3	N2—C16—H16A	109.5
C4—C5—H5	119.0	N2—C16—H16B	109.5
C6—C5—C4	121.9 (4)	N2—C16—H16C	109.5
C6—C5—H5	119.0	H16A—C16—H16B	109.5
C5—C6—H6	121.0	H16A—C16—H16C	109.5
C5—C6—C7	118.0 (3)	H16B—C16—H16C	109.5
C7—C6—H6	121.0	O3—C17—O4	124.6 (2)
N1—C7—C6	130.4 (3)	O3—C17—C18	120.1 (2)
N1—C7—C8	107.4 (3)	O4—C17—C18	115.3 (2)
C6—C7—C8	122.2 (3)	C17—C18—H18	118.0
C3—C8—C7	117.0 (3)	C19—C18—C17	124.0 (2)
C3—C8—C9	136.1 (3)	C19—C18—H18	118.0
C7—C8—C9	106.8 (3)	C18—C19—H19	118.6
C8—C9—C11	128.4 (3)	C18—C19—C20	122.8 (2)
C10—C9—C8	106.1 (3)	C20—C19—H19	118.6
C10—C9—C11	125.5 (3)	O5—C20—O6	125.1 (2)
N1—C10—C9	110.5 (3)	O5—C20—C19	121.3 (2)
N1—C10—H10	124.7	O6—C20—C19	113.6 (2)
			
O2—C3—C4—C5	−176.1 (3)	C6—C7—C8—C3	−1.0 (4)
O2—C3—C8—C7	176.5 (2)	C6—C7—C8—C9	179.9 (3)
O2—C3—C8—C9	−4.7 (5)	C7—N1—C10—C9	−0.9 (4)
O3—C17—C18—C19	−2.3 (6)	C7—C8—C9—C10	−0.1 (3)
O4—C17—C18—C19	176.4 (4)	C7—C8—C9—C11	177.7 (3)
N1—C7—C8—C3	178.7 (3)	C8—C3—C4—C5	−0.4 (5)
N1—C7—C8—C9	−0.4 (3)	C8—C9—C10—N1	0.6 (4)
N2—C13—C14—C15	125.8 (5)	C8—C9—C11—C12	142.7 (3)
C2—O2—C3—C4	−100.4 (3)	C9—C11—C12—N2	174.6 (3)
C2—O2—C3—C8	83.9 (4)	C10—N1—C7—C6	−179.6 (4)
C3—O2—C2—O1	−0.7 (5)	C10—N1—C7—C8	0.8 (4)
C3—O2—C2—C1	−180.0 (3)	C10—C9—C11—C12	−39.8 (4)
C3—C4—C5—C6	−0.1 (6)	C11—C9—C10—N1	−177.3 (3)
C3—C8—C9—C10	−179.0 (3)	C12—N2—C13—C14	−63.6 (4)
C3—C8—C9—C11	−1.1 (6)	C13—N2—C12—C11	158.9 (3)
C4—C3—C8—C7	0.9 (5)	C16—N2—C12—C11	−75.8 (4)
C4—C3—C8—C9	179.7 (3)	C16—N2—C13—C14	170.7 (3)
C4—C5—C6—C7	0.0 (6)	C17—C18—C19—C20	179.9 (3)
C5—C6—C7—N1	−179.0 (4)	C18—C19—C20—O5	−15.6 (5)
C5—C6—C7—C8	0.5 (5)	C18—C19—C20—O6	164.6 (3)

### [2-(4-Acetyloxy-1H-indol-3-yl)ethyl](methyl)prop-2-enylazanium 3-carboxyprop-2-enoate (II) Hydrogen-bond geometry (Å, º)

**Table:**

*D*—H···*A*	*D*—H	H···*A*	*D*···*A*	*D*—H···*A*
N1—H1···O5^i^	0.87 (1)	2.00 (2)	2.857 (3)	169 (4)
O4—H4*A*···O6^ii^	0.89 (1)	1.56 (2)	2.454 (3)	175 (6)

Symmetry codes: (i) *x*−2, *y*, *z*−1; (ii) *x*−1, *y*, *z*.

### Bis{[2-(4-acetyloxy-1H-indol-3-yl)ethyl]diprop-2-enylazanium} but-2-enedioate–(E)-butenedioic acid (1/1) (III) Crystal data

**Table:**

2C_18_H_23_N_2_O_2_^+^·C_4_H_2_O_4_^2^^−^·C_4_H_4_O_4_	*F*(000) = 1760
*M**_r_* = 828.90	*D*_x_ = 1.270 Mg m^−^^3^
Monoclinic, *C*2/*c*	Mo *K*α radiation, λ = 0.71073 Å
*a* = 23.6642 (19) Å	Cell parameters from 9906 reflections
*b* = 8.4204 (7) Å	θ = 2.6–24.9°
*c* = 23.4002 (18) Å	µ = 0.09 mm^−^^1^
β = 111.614 (2)°	*T* = 297 K
*V* = 4334.9 (6) Å^3^	Block, colorless
*Z* = 4	0.22 × 0.2 × 0.12 mm

### Bis{[2-(4-acetyloxy-1H-indol-3-yl)ethyl]diprop-2-enylazanium} but-2-enedioate–(E)-butenedioic acid (1/1) (III) Data collection

**Table:**

Bruker D8 Venture CMOS diffractometer	3441 reflections with *I* > 2σ(*I*)
φ and ω scans	*R*_int_ = 0.040
Absorption correction: multi-scan (SADABS; Bruker, 2018)	θ_max_ = 25.8°, θ_min_ = 2.6°
*T*_min_ = 0.715, *T*_max_ = 0.745	*h* = −28→28
99597 measured reflections	*k* = −10→10
4126 independent reflections	*l* = −28→28

### Bis{[2-(4-acetyloxy-1H-indol-3-yl)ethyl]diprop-2-enylazanium} but-2-enedioate–(E)-butenedioic acid (1/1) (III) Refinement

**Table:**

Refinement on *F*^2^	Hydrogen site location: mixed
Least-squares matrix: full	H atoms treated by a mixture of independent and constrained refinement
*R*[*F*^2^ > 2σ(*F*^2^)] = 0.051	*w* = 1/[σ^2^(*F*_o_^2^) + (0.0623*P*)^2^ + 3.3312*P*] where *P* = (*F*_o_^2^ + 2*F*_c_^2^)/3
*wR*(*F*^2^) = 0.140	(Δ/σ)_max_ = 0.001
*S* = 1.06	Δρ_max_ = 0.50 e Å^−^^3^
4126 reflections	Δρ_min_ = −0.29 e Å^−^^3^
354 parameters	Extinction correction: SHELXL2018 (Sheldrick, 2015b), Fc^*^=kFc[1+0.001xFc^2^λ^3^/sin(2θ)]^-1/4^
114 restraints	Extinction coefficient: 0.0057 (13)

### Bis{[2-(4-acetyloxy-1H-indol-3-yl)ethyl]diprop-2-enylazanium} but-2-enedioate–(E)-butenedioic acid (1/1) (III) Special details

**Table:**

Geometry. All esds (except the esd in the dihedral angle between two l.s. planes) are estimated using the full covariance matrix. The cell esds are taken into account individually in the estimation of esds in distances, angles and torsion angles; correlations between esds in cell parameters are only used when they are defined by crystal symmetry. An approximate (isotropic) treatment of cell esds is used for estimating esds involving l.s. planes.

### Bis{[2-(4-acetyloxy-1H-indol-3-yl)ethyl]diprop-2-enylazanium} but-2-enedioate–(E)-butenedioic acid (1/1) (III) Fractional atomic coordinates and isotropic or equivalent isotropic displacement parameters (Å^2^)

**Table:**

	*x*	*y*	*z*	*U*_iso_*/*U*_eq_	Occ. (<1)
O3	0.43377 (5)	0.84230 (16)	0.40787 (6)	0.0547 (4)	
O4	0.52519 (5)	0.82545 (19)	0.40300 (6)	0.0650 (4)	
N1	0.15205 (7)	0.5819 (2)	0.16972 (7)	0.0540 (4)	
H1	0.1284 (8)	0.542 (2)	0.1349 (6)	0.065*	
N2	0.40861 (6)	0.56750 (17)	0.34003 (6)	0.0444 (4)	
H2	0.4151 (9)	0.6559 (16)	0.3614 (8)	0.053*	
C3	0.19478 (8)	0.6147 (2)	0.33073 (8)	0.0533 (5)	
C4	0.14362 (9)	0.5733 (3)	0.34114 (10)	0.0683 (6)	
H4	0.144072	0.571251	0.381042	0.082*	
C5	0.09055 (9)	0.5341 (3)	0.29169 (12)	0.0705 (6)	
H5	0.055901	0.506800	0.299318	0.085*	
C6	0.08822 (8)	0.5348 (2)	0.23271 (10)	0.0590 (5)	
H6	0.052601	0.509456	0.200080	0.071*	
C7	0.14110 (7)	0.5749 (2)	0.22293 (8)	0.0467 (4)	
C8	0.19553 (7)	0.61618 (19)	0.27159 (8)	0.0432 (4)	
C9	0.24029 (7)	0.6461 (2)	0.24481 (8)	0.0450 (4)	
C10	0.21169 (8)	0.6216 (2)	0.18357 (9)	0.0537 (5)	
H10	0.230151	0.630485	0.154837	0.064*	
C11	0.30631 (7)	0.6867 (2)	0.27697 (9)	0.0496 (4)	
H11A	0.310383	0.771998	0.306092	0.060*	
H11B	0.323061	0.722895	0.247150	0.060*	
C12	0.34109 (7)	0.5424 (2)	0.31047 (9)	0.0480 (4)	
H12A	0.325670	0.511823	0.341969	0.058*	
H12B	0.333443	0.455047	0.281562	0.058*	
C16	0.43581 (9)	0.4308 (3)	0.38317 (11)	0.0664 (6)	
H16A	0.417335	0.427499	0.413909	0.080*	
H16B	0.425604	0.332482	0.360012	0.080*	
C17	0.50234 (11)	0.4390 (3)	0.41471 (13)	0.0871 (8)	
H17	0.519123	0.527455	0.438803	0.105*	
C18	0.53850 (14)	0.3276 (5)	0.41018 (19)	0.1316 (14)	
H18A	0.522600	0.238260	0.386311	0.158*	
H18B	0.580261	0.337274	0.430818	0.158*	
C19	0.48981 (7)	0.8722 (2)	0.42812 (8)	0.0467 (4)	
C20	0.51576 (7)	0.9709 (2)	0.48471 (8)	0.0479 (4)	
H20	0.557205	0.992472	0.499179	0.057*	
O5	0.6457 (5)	0.730 (3)	0.5379 (4)	0.097 (5)	0.48 (4)
O6	0.6402 (8)	0.822 (2)	0.4447 (8)	0.062 (2)	0.48 (4)
H6A	0.603390	0.828442	0.436178	0.094*	0.48 (4)
C21	0.6647 (8)	0.748 (3)	0.4963 (7)	0.060 (3)	0.48 (4)
O5A	0.6392 (3)	0.645 (2)	0.5186 (10)	0.103 (4)	0.52 (4)
O6A	0.6418 (7)	0.800 (3)	0.4512 (8)	0.084 (4)	0.52 (4)
H6AA	0.605462	0.793944	0.444863	0.126*	0.52 (4)
C21A	0.6724 (9)	0.727 (3)	0.5011 (8)	0.070 (4)	0.52 (4)
C22	0.73480 (9)	0.7230 (3)	0.51577 (9)	0.0637 (6)	
H22	0.755398	0.666681	0.551626	0.076*	0.48 (4)
H22A	0.757554	0.675587	0.553077	0.076*	0.52 (4)
C13	0.43825 (8)	0.5987 (3)	0.29456 (9)	0.0579 (5)	
H13A	0.470397	0.672837	0.316182	0.069*	0.099 (4)
H13B	0.458837	0.499336	0.294117	0.069*	0.099 (4)
H13C	0.427809	0.705354	0.278487	0.069*	0.901 (4)
H13D	0.481992	0.594839	0.315899	0.069*	0.901 (4)
C14	0.4188 (10)	0.652 (3)	0.2307 (5)	0.0769 (8)	0.099 (4)
H14	0.423405	0.761671	0.228839	0.092*	0.099 (4)
C15	0.397 (3)	0.592 (7)	0.1760 (14)	0.1028 (15)	0.099 (4)
H15A	0.390253	0.483222	0.170924	0.123*	0.099 (4)
H15B	0.388512	0.657311	0.141789	0.123*	0.099 (4)
C14A	0.42144 (12)	0.4880 (3)	0.24310 (12)	0.0769 (8)	0.901 (4)
H14A	0.428767	0.380233	0.251180	0.092*	0.901 (4)
C15A	0.3967 (3)	0.5342 (6)	0.18674 (15)	0.1028 (15)	0.901 (4)
H15C	0.389088	0.641456	0.177808	0.123*	0.901 (4)
H15D	0.386609	0.460156	0.155046	0.123*	0.901 (4)
O1	0.2254 (8)	0.8833 (10)	0.3924 (13)	0.099 (6)	0.38 (4)
O2	0.2527 (6)	0.645 (2)	0.3753 (9)	0.071 (5)	0.38 (4)
C1	0.3155 (7)	0.789 (2)	0.4602 (7)	0.066 (4)	0.38 (4)
H1A	0.333536	0.689257	0.477037	0.098*	0.38 (4)
H1B	0.313766	0.856311	0.492675	0.098*	0.38 (4)
H1C	0.339598	0.839519	0.440153	0.098*	0.38 (4)
C2	0.2529 (8)	0.7621 (19)	0.4148 (8)	0.064 (6)	0.38 (4)
O1A	0.2133 (4)	0.848 (2)	0.4172 (7)	0.102 (4)	0.62 (4)
O2A	0.2484 (4)	0.6422 (12)	0.3825 (6)	0.063 (2)	0.62 (4)
C1A	0.3206 (6)	0.772 (2)	0.4660 (6)	0.113 (5)	0.62 (4)
H1AA	0.350811	0.753864	0.448371	0.170*	0.62 (4)
H1AB	0.321755	0.687720	0.493898	0.170*	0.62 (4)
H1AC	0.328772	0.871404	0.487805	0.170*	0.62 (4)
C2A	0.2590 (5)	0.7784 (14)	0.4159 (6)	0.071 (4)	0.62 (4)

### Bis{[2-(4-acetyloxy-1H-indol-3-yl)ethyl]diprop-2-enylazanium} but-2-enedioate–(E)-butenedioic acid (1/1) (III) Atomic displacement parameters (Å^2^)

**Table:**

	*U*^11^	*U*^22^	*U*^33^	*U*^12^	*U*^13^	*U*^23^
O3	0.0303 (6)	0.0649 (8)	0.0582 (7)	−0.0035 (5)	0.0039 (5)	−0.0155 (6)
O4	0.0370 (7)	0.0920 (11)	0.0605 (8)	−0.0001 (6)	0.0115 (6)	−0.0220 (7)
N1	0.0411 (8)	0.0576 (9)	0.0510 (9)	0.0042 (7)	0.0024 (6)	−0.0055 (7)
N2	0.0326 (7)	0.0479 (8)	0.0502 (8)	−0.0044 (6)	0.0122 (6)	−0.0050 (6)
C3	0.0375 (9)	0.0627 (11)	0.0553 (10)	0.0019 (8)	0.0119 (8)	−0.0016 (9)
C4	0.0538 (11)	0.0892 (16)	0.0675 (13)	−0.0008 (11)	0.0288 (10)	0.0034 (11)
C5	0.0405 (10)	0.0807 (15)	0.0945 (16)	−0.0076 (10)	0.0299 (11)	−0.0024 (12)
C6	0.0314 (8)	0.0566 (11)	0.0806 (14)	−0.0009 (8)	0.0108 (8)	−0.0072 (10)
C7	0.0343 (8)	0.0396 (9)	0.0584 (10)	0.0039 (6)	0.0079 (7)	−0.0025 (7)
C8	0.0313 (8)	0.0403 (8)	0.0528 (9)	0.0026 (6)	0.0095 (7)	−0.0019 (7)
C9	0.0348 (8)	0.0417 (9)	0.0553 (10)	0.0021 (7)	0.0128 (7)	−0.0009 (7)
C10	0.0468 (10)	0.0573 (11)	0.0553 (10)	0.0048 (8)	0.0170 (8)	0.0018 (8)
C11	0.0353 (8)	0.0457 (9)	0.0664 (11)	−0.0047 (7)	0.0170 (8)	−0.0052 (8)
C12	0.0307 (8)	0.0488 (9)	0.0624 (10)	−0.0065 (7)	0.0147 (7)	−0.0048 (8)
C16	0.0520 (11)	0.0612 (12)	0.0793 (14)	−0.0024 (9)	0.0163 (10)	0.0168 (10)
C17	0.0572 (13)	0.0821 (16)	0.0994 (18)	−0.0041 (12)	0.0022 (12)	0.0325 (14)
C18	0.0713 (18)	0.136 (3)	0.169 (4)	0.0369 (19)	0.021 (2)	0.068 (3)
C19	0.0323 (8)	0.0518 (10)	0.0467 (9)	0.0012 (7)	0.0035 (7)	−0.0023 (7)
C20	0.0288 (7)	0.0548 (10)	0.0505 (9)	−0.0047 (7)	0.0033 (6)	−0.0049 (8)
O5	0.053 (3)	0.178 (11)	0.073 (3)	0.018 (5)	0.037 (2)	0.035 (4)
O6	0.039 (4)	0.086 (4)	0.055 (4)	0.004 (3)	0.009 (3)	0.024 (3)
C21	0.021 (3)	0.104 (9)	0.054 (4)	0.014 (4)	0.014 (3)	0.030 (5)
O5A	0.055 (2)	0.139 (8)	0.120 (7)	−0.005 (3)	0.039 (3)	0.053 (6)
O6A	0.033 (4)	0.140 (11)	0.079 (6)	0.007 (4)	0.021 (4)	0.032 (5)
C21A	0.051 (8)	0.085 (5)	0.073 (6)	0.012 (5)	0.021 (5)	0.019 (5)
C22	0.0401 (10)	0.0906 (15)	0.0582 (11)	0.0058 (10)	0.0156 (8)	0.0138 (11)
C13	0.0387 (9)	0.0753 (13)	0.0602 (11)	−0.0093 (9)	0.0190 (8)	−0.0079 (10)
C14	0.0792 (17)	0.0819 (17)	0.0850 (19)	−0.0242 (14)	0.0481 (15)	−0.0265 (14)
C15	0.0831 (18)	0.165 (4)	0.0664 (19)	−0.026 (3)	0.0341 (17)	−0.027 (2)
C14A	0.0792 (17)	0.0819 (17)	0.0850 (19)	−0.0242 (14)	0.0481 (15)	−0.0265 (14)
C15A	0.0831 (18)	0.165 (4)	0.0664 (19)	−0.026 (3)	0.0341 (17)	−0.027 (2)
O1	0.087 (5)	0.069 (4)	0.116 (10)	−0.002 (3)	0.006 (6)	−0.008 (4)
O2	0.043 (4)	0.126 (10)	0.045 (4)	−0.007 (5)	0.015 (3)	−0.025 (5)
C1	0.052 (7)	0.099 (8)	0.046 (5)	−0.009 (6)	0.018 (6)	−0.016 (5)
C2	0.040 (6)	0.086 (11)	0.048 (7)	−0.005 (6)	−0.005 (5)	0.004 (6)
O1A	0.076 (3)	0.111 (6)	0.101 (5)	0.016 (3)	0.013 (3)	−0.032 (4)
O2A	0.040 (2)	0.090 (4)	0.052 (4)	0.012 (3)	0.009 (2)	−0.005 (3)
C1A	0.067 (5)	0.168 (12)	0.075 (5)	−0.018 (6)	−0.009 (4)	−0.013 (6)
C2A	0.066 (6)	0.079 (6)	0.070 (6)	−0.022 (4)	0.027 (5)	−0.014 (4)

### Bis{[2-(4-acetyloxy-1H-indol-3-yl)ethyl]diprop-2-enylazanium} but-2-enedioate–(E)-butenedioic acid (1/1) (III) Geometric parameters (Å, º)

**Table:**

O3—C19	1.2583 (19)	C20—H20	0.9300
O4—C19	1.251 (2)	O5—C21	1.223 (9)
N1—H1	0.868 (10)	O6—H6A	0.8200
N1—C7	1.363 (2)	O6—C21	1.289 (8)
N1—C10	1.368 (2)	C21—C22	1.564 (18)
N2—H2	0.878 (9)	O5A—C21A	1.221 (10)
N2—C12	1.5038 (19)	O6A—H6AA	0.8200
N2—C16	1.510 (2)	O6A—C21A	1.285 (8)
N2—C13	1.498 (2)	C21A—C22	1.388 (19)
C3—C4	1.365 (3)	C22—C22^ii^	1.288 (4)
C3—C8	1.391 (3)	C22—H22	0.9300
C3—O2	1.407 (9)	C22—H22A	0.9300
C3—O2A	1.413 (6)	C13—H13A	0.9700
C4—H4	0.9300	C13—H13B	0.9700
C4—C5	1.398 (3)	C13—H13C	0.9700
C5—H5	0.9300	C13—H13D	0.9700
C5—C6	1.361 (3)	C13—C14	1.465 (10)
C6—H6	0.9300	C13—C14A	1.458 (3)
C6—C7	1.394 (3)	C14—H14	0.9300
C7—C8	1.413 (2)	C14—C15	1.294 (10)
C8—C9	1.438 (2)	C15—H15A	0.9300
C9—C10	1.357 (3)	C15—H15B	0.9300
C9—C11	1.503 (2)	C14A—H14A	0.9300
C10—H10	0.9300	C14A—C15A	1.290 (4)
C11—H11A	0.9700	C15A—H15C	0.9300
C11—H11B	0.9700	C15A—H15D	0.9300
C11—C12	1.514 (2)	O1—C2	1.220 (10)
C12—H12A	0.9700	O2—C2	1.348 (9)
C12—H12B	0.9700	C1—H1A	0.9600
C16—H16A	0.9700	C1—H1B	0.9600
C16—H16B	0.9700	C1—H1C	0.9600
C16—C17	1.474 (3)	C1—C2	1.489 (9)
C17—H17	0.9300	O1A—C2A	1.239 (9)
C17—C18	1.300 (4)	O2A—C2A	1.359 (7)
C18—H18A	0.9300	C1A—H1AA	0.9600
C18—H18B	0.9300	C1A—H1AB	0.9600
C19—C20	1.491 (2)	C1A—H1AC	0.9600
C20—C20^i^	1.304 (3)	C1A—C2A	1.498 (8)
			
C7—N1—H1	124.6 (14)	C19—C20—H20	117.8
C7—N1—C10	108.71 (15)	C20^i^—C20—C19	124.40 (19)
C10—N1—H1	124.6 (14)	C20^i^—C20—H20	117.8
C12—N2—H2	108.3 (13)	C21—O6—H6A	109.5
C12—N2—C16	108.62 (13)	O5—C21—O6	129.4 (19)
C16—N2—H2	108.9 (13)	O5—C21—C22	114.3 (14)
C13—N2—H2	103.1 (13)	O6—C21—C22	113.7 (10)
C13—N2—C12	113.22 (14)	C21A—O6A—H6AA	109.5
C13—N2—C16	114.38 (15)	O5A—C21A—O6A	111 (2)
C4—C3—C8	121.14 (17)	O5A—C21A—C22	131.5 (18)
C4—C3—O2	126.9 (12)	O6A—C21A—C22	115.3 (11)
C4—C3—O2A	117.6 (8)	C21—C22—H22	118.6
C8—C3—O2	111.7 (12)	C21A—C22—H22A	115.9
C8—C3—O2A	121.0 (8)	C22^ii^—C22—C21	122.7 (5)
C3—C4—H4	120.1	C22^ii^—C22—C21A	128.2 (7)
C3—C4—C5	119.9 (2)	C22^ii^—C22—H22	118.6
C5—C4—H4	120.1	C22^ii^—C22—H22A	115.9
C4—C5—H5	119.1	N2—C13—H13A	103.0
C6—C5—C4	121.79 (18)	N2—C13—H13B	103.0
C6—C5—H5	119.1	N2—C13—H13C	108.6
C5—C6—H6	121.2	N2—C13—H13D	108.6
C5—C6—C7	117.65 (18)	H13A—C13—H13B	105.1
C7—C6—H6	121.2	H13C—C13—H13D	107.6
N1—C7—C6	130.13 (17)	C14—C13—N2	136.6 (9)
N1—C7—C8	107.57 (15)	C14—C13—H13A	103.0
C6—C7—C8	122.29 (18)	C14—C13—H13B	103.0
C3—C8—C7	117.24 (15)	C14A—C13—N2	114.71 (17)
C3—C8—C9	135.79 (15)	C14A—C13—H13C	108.6
C7—C8—C9	106.93 (15)	C14A—C13—H13D	108.6
C8—C9—C11	128.22 (16)	C13—C14—H14	110.7
C10—C9—C8	105.82 (15)	C15—C14—C13	139 (3)
C10—C9—C11	125.87 (16)	C15—C14—H14	110.7
N1—C10—H10	124.5	C14—C15—H15A	120.0
C9—C10—N1	110.92 (16)	C14—C15—H15B	120.0
C9—C10—H10	124.5	H15A—C15—H15B	120.0
C9—C11—H11A	109.6	C13—C14A—H14A	118.8
C9—C11—H11B	109.6	C15A—C14A—C13	122.3 (3)
C9—C11—C12	110.08 (14)	C15A—C14A—H14A	118.8
H11A—C11—H11B	108.2	C14A—C15A—H15C	120.0
C12—C11—H11A	109.6	C14A—C15A—H15D	120.0
C12—C11—H11B	109.6	H15C—C15A—H15D	120.0
N2—C12—C11	114.08 (14)	C2—O2—C3	113.5 (14)
N2—C12—H12A	108.7	H1A—C1—H1B	109.5
N2—C12—H12B	108.7	H1A—C1—H1C	109.5
C11—C12—H12A	108.7	H1B—C1—H1C	109.5
C11—C12—H12B	108.7	C2—C1—H1A	109.5
H12A—C12—H12B	107.6	C2—C1—H1B	109.5
N2—C16—H16A	108.7	C2—C1—H1C	109.5
N2—C16—H16B	108.7	O1—C2—O2	117 (2)
H16A—C16—H16B	107.6	O1—C2—C1	114.3 (18)
C17—C16—N2	114.37 (17)	O2—C2—C1	110.6 (12)
C17—C16—H16A	108.7	C2A—O2A—C3	123.3 (8)
C17—C16—H16B	108.7	H1AA—C1A—H1AB	109.5
C16—C17—H17	118.7	H1AA—C1A—H1AC	109.5
C18—C17—C16	122.6 (3)	H1AB—C1A—H1AC	109.5
C18—C17—H17	118.7	C2A—C1A—H1AA	109.5
C17—C18—H18A	120.0	C2A—C1A—H1AB	109.5
C17—C18—H18B	120.0	C2A—C1A—H1AC	109.5
H18A—C18—H18B	120.0	O1A—C2A—O2A	115.7 (14)
O3—C19—C20	118.57 (15)	O1A—C2A—C1A	127.1 (11)
O4—C19—O3	123.72 (16)	O2A—C2A—C1A	110.0 (9)
O4—C19—C20	117.70 (14)		
			
O3—C19—C20—C20^i^	0.2 (3)	C8—C3—O2—C2	128.5 (19)
O4—C19—C20—C20^i^	179.3 (2)	C8—C3—O2A—C2A	107.5 (16)
N1—C7—C8—C3	−179.21 (16)	C8—C9—C10—N1	1.3 (2)
N1—C7—C8—C9	−0.90 (18)	C8—C9—C11—C12	71.7 (2)
N2—C16—C17—C18	−121.8 (3)	C9—C11—C12—N2	175.40 (14)
N2—C13—C14—C15	−86 (5)	C10—N1—C7—C6	−177.82 (19)
N2—C13—C14A—C15A	121.5 (3)	C10—N1—C7—C8	1.7 (2)
C3—C4—C5—C6	0.4 (4)	C10—C9—C11—C12	−104.3 (2)
C3—C8—C9—C10	177.6 (2)	C11—C9—C10—N1	178.07 (16)
C3—C8—C9—C11	1.0 (3)	C12—N2—C16—C17	179.1 (2)
C3—O2—C2—O1	−47 (4)	C12—N2—C13—C14	20.0 (14)
C3—O2—C2—C1	−179.5 (18)	C12—N2—C13—C14A	−48.3 (2)
C3—O2A—C2A—O1A	29 (3)	C16—N2—C12—C11	167.33 (16)
C3—O2A—C2A—C1A	−177.9 (14)	C16—N2—C13—C14	145.2 (14)
C4—C3—C8—C7	0.7 (3)	C16—N2—C13—C14A	76.8 (2)
C4—C3—C8—C9	−177.0 (2)	O5—C21—C22—C22^ii^	−160.9 (18)
C4—C3—O2—C2	−57 (3)	O6—C21—C22—C22^ii^	3 (2)
C4—C3—O2A—C2A	−78.0 (17)	O5A—C21A—C22—C22^ii^	158 (2)
C4—C5—C6—C7	0.6 (3)	O6A—C21A—C22—C22^ii^	−4 (3)
C5—C6—C7—N1	178.49 (19)	C13—N2—C12—C11	−64.5 (2)
C5—C6—C7—C8	−1.0 (3)	C13—N2—C16—C17	51.5 (3)
C6—C7—C8—C3	0.4 (3)	O2—C3—C4—C5	−174.8 (10)
C6—C7—C8—C9	178.68 (16)	O2—C3—C8—C7	175.3 (9)
C7—N1—C10—C9	−2.0 (2)	O2—C3—C8—C9	−2.4 (9)
C7—C8—C9—C10	−0.25 (19)	O2A—C3—C4—C5	−175.5 (5)
C7—C8—C9—C11	−176.89 (16)	O2A—C3—C8—C7	175.0 (5)
C8—C3—C4—C5	−1.1 (3)	O2A—C3—C8—C9	−2.7 (6)

Symmetry codes: (i) −*x*+1, −*y*+2, −*z*+1; (ii) −*x*+3/2, −*y*+3/2, −*z*+1.

### Bis{[2-(4-acetyloxy-1H-indol-3-yl)ethyl]diprop-2-enylazanium} but-2-enedioate–(E)-butenedioic acid (1/1) (III) Hydrogen-bond geometry (Å, º)

**Table:**

*D*—H···*A*	*D*—H	H···*A*	*D*···*A*	*D*—H···*A*
N1—H1···O3^iii^	0.87 (1)	2.22 (2)	2.962 (2)	144 (2)
N2—H2···O3	0.88 (1)	1.87 (1)	2.7446 (19)	177 (2)
O6—H6*A*···O4	0.82	1.72	2.530 (17)	168
O6*A*—H6*AA*···O4	0.82	1.81	2.577 (16)	156

Symmetry code: (iii) −*x*+1/2, *y*−1/2, −*z*+1/2.
